# Genetic diversity and population structure of eddoe taro in China using genome-wide SNP markers

**DOI:** 10.7717/peerj.10485

**Published:** 2020-12-08

**Authors:** Zhixin Wang, Yalin Sun, Xinfang Huang, Feng Li, Yuping Liu, Honglian Zhu, Zhengwei Liu, Weidong Ke

**Affiliations:** Wuhan Academy of Agricultural Sciences, Wuhan, Hubei, China

**Keywords:** Colocasia esculenta, Genetic diversity, Population structure, Core germplasm, SNP

## Abstract

Taro (*Colocasia esculenta*) is an important root and tuber crop cultivated worldwide. There are two main types of taro that vary in morphology of corm and cormel, ‘dasheen’ and ‘eddoe’. The eddoe type (*Colocasia esculenta var. antiquorium*) is predominantly distributed throughout China. Characterizing the genetic diversity present in the germplasm bank of taro is fundamental to better manage, conserve and utilize the genetic resources of this species. In this study, the genetic diversity of 234 taro accessions from 16 provinces of China was assessed using 132,869 single nucleotide polymorphism (SNP) markers identified by specific length amplified fragment-sequencing (SLAF-seq). Population structure and principal component analysis permitted the accessions to be categorized into eight groups. The genetic diversity and population differentiation of the eight groups were evaluated using the characterized SNPs. Analysis of molecular variance showed that the variation among eight inferred groups was higher than that within groups, while a relatively small variance was found among the two morphological types and 16 collection regions. Further, a core germplasm set comprising 41 taro accessions that maintained the genetic diversity of the entire collection was developed based on the genotype. This research is expected to be valuable for genetic characterization, germplasm conservation, and breeding of taro.

## Introduction

Taro (*Colocasia esculenta*), a member of the *Araceae* family, monocotyledons, is one of the most important root and tuber crops. It is consumed as a staple food and vegetable by more than 500 million people in regions of the Asian-Pacific, Africa, and America (Simsek & Nehir El, 2015). The underground corms and cormels of taro, the major edible parts, have a high nutritional value and are rich in a range of health-related compounds that protect against a variety of human diseases (Alcantara, Hurtada & Dizon, 2013; Pereira et al., 2018; Simsek & Nehir El, 2015).

Taro is believed to have been domesticated, possibly independently, across an area that ranges from northeastern India to the province of Yunnan in China, to New Guinea (Miyasaka et al., 2019). China has a long history of taro cultivation for more than two thousand years, and is the second largest producer and the largest exporter of taro in the world (FAOSTAT, 2020; UNcomtrade, 2020). Thus, China has a rich diversity of landraces and wild relatives of taro. Based on the ethnobotanical, morphological, and genetic characterization, Xu et al. (2001) divided Chinese taro into five morphotypes: inflorescence, single corm, multi-cormel, multi-corm, and petiole type. Furthermore, they proposed that the multi-corm type was domesticated from the multi-cormel type; the latter has a small, globular corm with relatively large cormels and is almost identical to the ‘eddoe’ type (*Colocasia esculenta var. antiquorium*). Cytological studies using flow cytometry analysis showed that taro has diploid (2 ×  = 2*n* = 28) as well as triploid (3 ×  = 3*n* = 42) cytotypes. Huang et al. (2012) found that the single-corm type was diploid, while the multi-cormel and multi-corm type were triploid. Due to better adaptability to geographical and climatic conditions, the triploid taro predominantly distributes in China. According to the color of petiole and corm bud, multi-cormel and multi-corm type taro were further divided into five subtypes (Huang et al., 2016).

As vegetative propagation and fixation of somatic mutations are common in taro, cultivars can be quite distinct in morphotype even within the same genetic background (Kuruvilla & Singh, 1981). Further, taro cultivars likely spread in many different directions over vast distances with trade and human migration (Matthews & Nguyen, 2014). These characteristics of taro make selection breeding by merely morphological characterization difficult. Therefore, molecular markers have been widely applied in the genetic characterization for taro. Thus, Irwin et al. (1998) utilized random amplification of polymorphic DNA (RAPD) markers to evaluate the genetic diversity of 44 taro accessions, and found that the taro accessions from Indonesia showed high genetic diversity. Similarly, Shen et al. (2003) analyzed the genetic diversity of 28 taro accessions collected from Yunnan province of China with isozymes, RAPD and amplified fragment length polymorphism (AFLP) markers, and found significant genetic diversity among these accessions. In another study, 10 simple sequence repeat (SSR) markers were used to categorize 22 taro cultivars from North East India into two clusters (Khatemenla et al., 2019).

In the absence of a reference genome, various kinds of next-generation sequencing strategies such as genotype by sequencing (GBS) (Shintaku et al., 2016; Helmkampf et al., 2017; Soulard, Mournet & Guitton, 2017) and RNA-seq (Liu et al., 2015; You et al., 2015; Wang et al., 2017b) have been adopted for development of genome-wide molecular markers for taro. For example, using GBS, Soulard, Mournet & Guitton (2017) constructed two unprecedented genetic linkage maps of taro with filtered single nucleotide polymorphisms (SNPs) and 14 SSR markers; in turn, Helmkampf et al. (2017) developed over 2400 high quality SNPs from 70 taro accessions, and uncovered the underlying phylogenetic relationships between Hawaiian and other Pacific landraces. Further, Wang et al. (2017b) identified 11,363 candidate SSR markers through the transcriptome sequencing of the taro variety “Jingjiang Xiangsha”. Specific length amplified fragment-sequencing (SLAF-seq), a kind of high-throughput genotyping method similar to GBS that based on next generation sequencing technology that does not require a reference genome (Sun et al., 2013), has been widely employed to identify genome-wide SNPs in large plant populations (Xu et al., 2014; Su et al., 2017; Wu et al., 2019).

A core germplasm collection is defined as a limited set of accessions representing the genetic diversity of a crop species (Frankel, 1984). As taro is an asexually propagated plant that almost sets no seeds under natural cultivation, a large size of germplasm collection hinders its conservation and management. Besides, the development of a representative core collection with maximum genetic diversity will help to use genetic accessions efficiently in taro breeding programs.

Despite phenotypic diversity, there is little information on the genetic diversity within the Chinese taro collection based on genome-wide molecular characterization. Exploring the genetic diversity contained in the taro collection will contribute to the enhanced genetic characterization, germplasm resources conservation and introduction breeding of this species. Therefore, this study aims to characterize the population structure and genetic diversity of 234 taro accessions comprising the collections maintained in the National Germplasm Repository for Aquatic Vegetable of China, and to develop a core collection maintaining most of the genetic diversity using the genome-wide SNP identified by the SLAF-seq.

## Materials & Methods

### Plant varieties and DNA extraction

**Figure 1:**
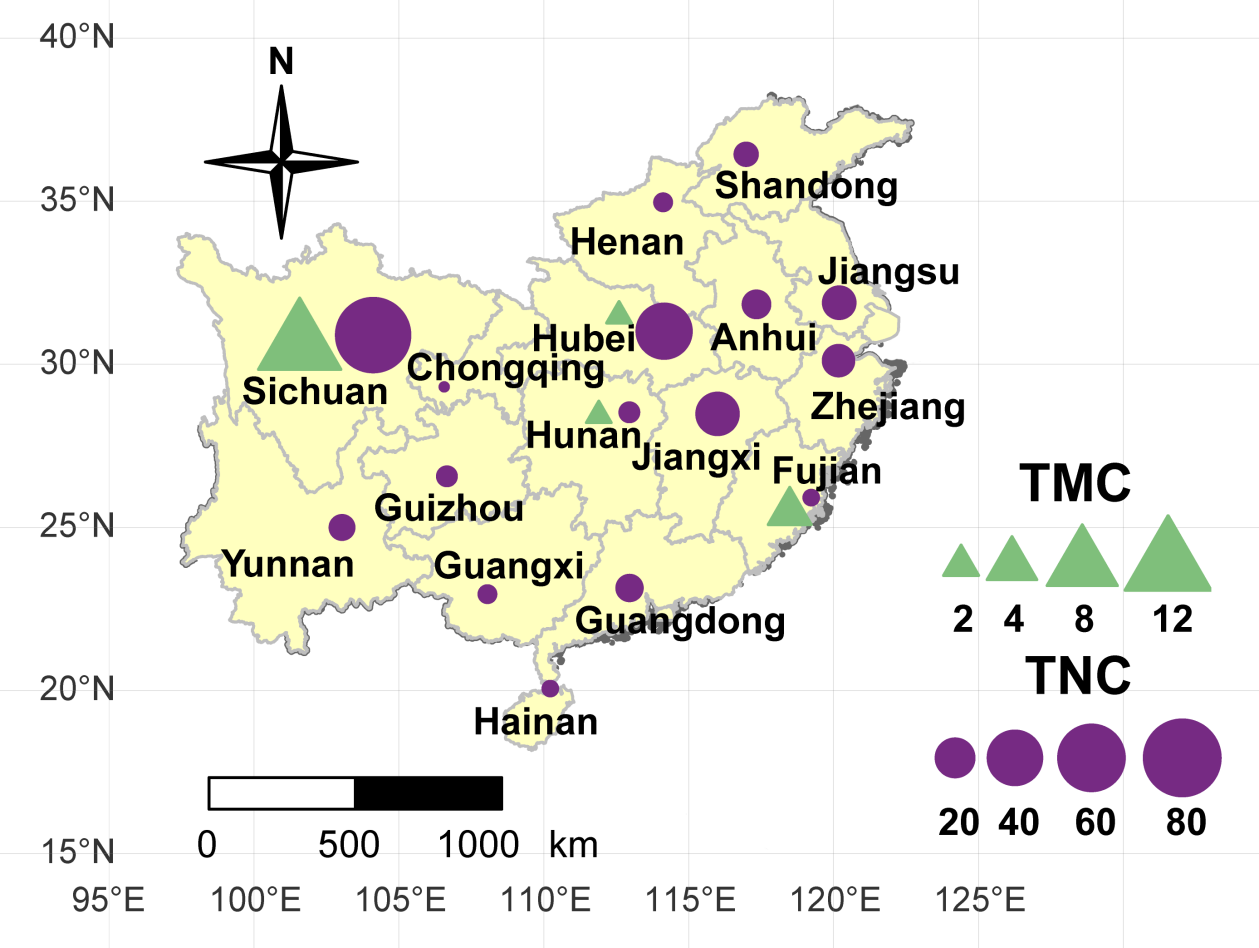
Geographical location of the taro accessions used in present study. The sizes of the purple circles and green triangles indicate the sample sizes of multi-cormel (TNC) and multi-corm (TMC) type taro in the collecting regions, respectively.

**Figure 2:**
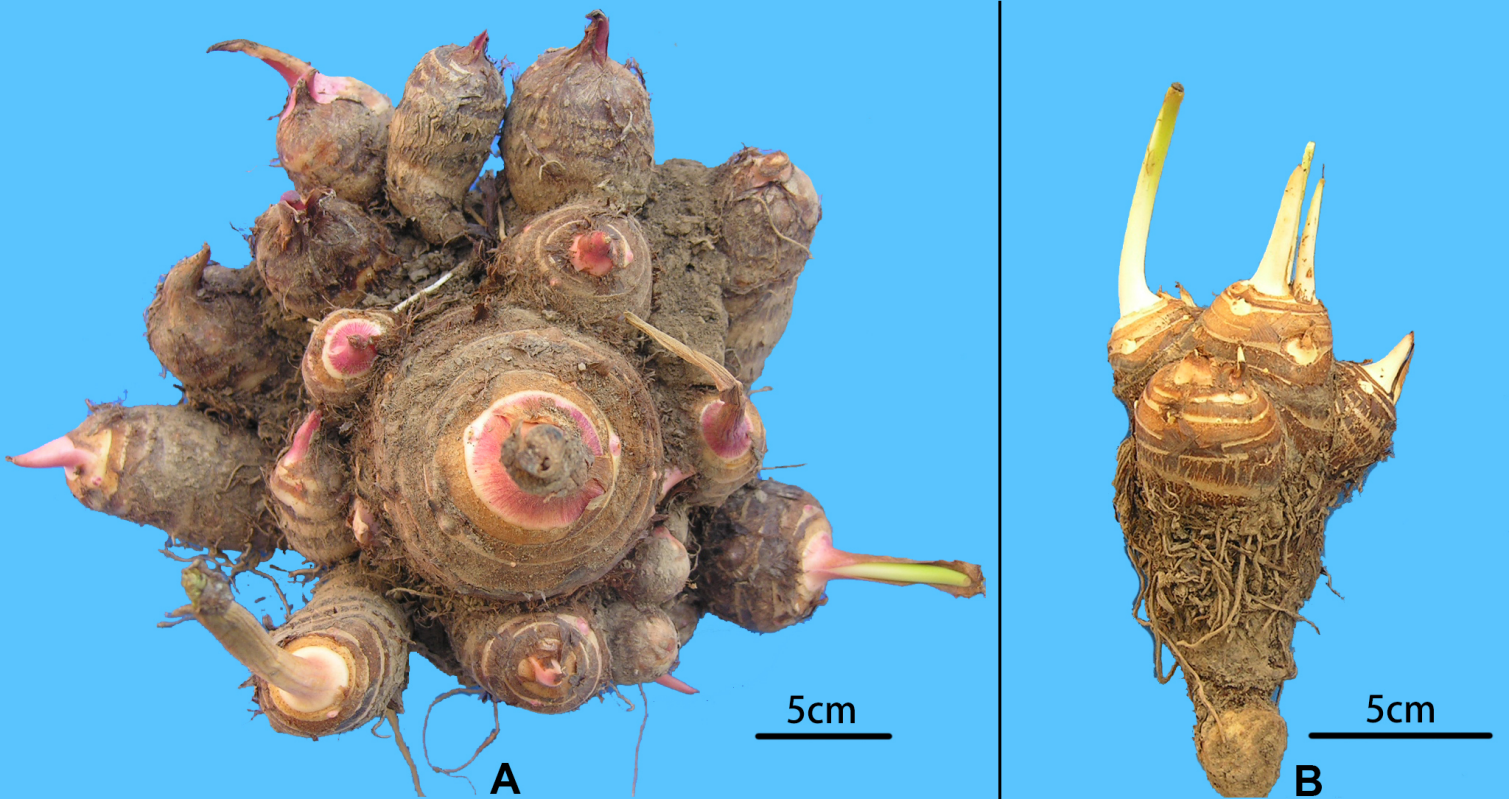
Morphological comparison of multi-cormel and multi-corm type. (A) Multi-cormel taro with red bud; (B) multi-corm taro with white bud.

A collection of 234 taro accessions from 16 different provinces of China, including 16 multi-corm type and 218 multi-cormel type (Fig. 1, Table S1), were selected for SNP identification in the present study. The multi-corm and multi-cormel types of taro can be distinguished by the morphology of their underground corms and cormels (Fig. 2). These accessions were cultivated in separate rows with plant spacing of 50 cm and labeled with the accession name to avoid mechanical mixture on the experimental farm of the National Germplasm Repository for Aquatic Vegetable at Jiangxia District, Wuhan, China (30°20′, 114°14′; at an elevation of 32 m). Fresh healthy leaves from each taro accession were sampled, frozen in liquid nitrogen immediately and stored at −80 °C until DNA extraction. DNA was extracted according to the CTAB method. The concentration and quality of DNA samples was quantified with a NanoDrop-2000 spectrophotometer, and DNA samples were adjusted to 50 ng/µL.

### SLAF-seq library preparation and sequencing

The SLAF library was perpared based on the procedures described by Sun et al. (2013) with some modifications. Genomic DNA of the accessions was digested with restriction enzymes HaeIII and EcoRV, with rice (Oryza sativa cv Nipponbare) DNA as a control to evaluate the efficiency of enzyme digestion. DNA fragments that were 364–414 bp (with indexes and adaptors) in length were chosen as SLAF tags to prepare paired-end (2 × 125 bp) sequencing with the Illumina High-Seq2500 system (Illumina, Inc.; San Diego, CA, US) at Beijing Biomarker Technologies Corporation.

### Raw read processing, SNP calling and filtering

After filtering out adapter reads and low-quality reads, the sequencing quality was then evaluated by calculating the guanine-cytosine (GC) content and the Q30 value (Table S2). The clean reads were uploaded to NCBI SRA database (http://www.ncbi.nlm.nih.gov/sra/) under the BioProject ID PRJNA644023. Sequences of sample with the most tags were used as the reference. BWA 0.7.17 (Li & Durbin, 2009) was used to align reads to the reference. GATK 3.7.0 (McKenna et al., 2010) and SAMtools 1.9 (Li et al., 2009) were used for SNP calling. And the intersection of SNPs called by GATK and SAMtools were considered as potential high-quality SNPs. Samples with a coverage less than 10 folds were removed, and SNPs with >0.2 missing data rate and minor allele frequency (MAF) <0.05 were removed by VCFtools 0.1.16 (Danecek et al., 2011). After that, 132,869 high-quality SNPs (Table S3) were identified, which were used to perform the following analyses.

### Population structure, principal component analysis and maximum likelihood (ML) tree

Before running ADMIXTURE, SNP data set was screened by Hardy-Weinberg equilibrium (HWE), linkage disequilibrium (LD) and outlier loci. SNPs with *p* value of HWE less than 0.0001 and LD decay with r^2^ great than 0.1 were filtered out through PLINK v1.90b4 (Purcell et al., 2007), and outlier loci were detected by BayeScan v2.1 (Fischer et al., 2011) and Lositan (Antao et al., 2008) with default settings. Population structure was estimated by ADMIXTURE v1.3.0 (Alexander, Novembre & Lange, 2009) with ten independent simulations for each K ranging from 1 to 10. A 10-fold cross-validation (CV) procedure was performed in order to calculate the CV errors for each K, and the value of K that exhibited a lowest CV error was selected as the optimal number of populations. Additionally, all the accessions were assigned to a corresponding group based on their ancestry fractions. The R package ‘pophelper’ that implements CLUMPP was used to plot the population structure (Francis, 2017).

To complement the results from ADMIXTURE, the DAPC function of the “adegenet” R package (Jombart, 2008) was used to determine the optimal number of clusters and assign individual accessions to the different clusters with the 132,869 high-quality SNPs. The optimal number of clusters from DAPC were determined by the K value corresponding to the lowest Bayesian Information Criterion (BIC). Thereafter DAPC clustering was performed on the clusters identified using the 40 principal components. Principal component analysis (PCA) was performed with TASSEL v.5 (Bradbury et al., 2007). A maximum likelihood (ML) phylogenetic tree was produced using RAxML-NG v.1.0.0 (Kozlov et al., 2019) under the GTRGAMMA model with 200 bootstrap replicates, and the best ML tree was displayed through iTOL v4 (https://itol.embl.de/) (Letunic & Bork, 2019).

### Genetic diversity and development of core germplasm

Nei’s genetic diversity index (H), polymorphic information content (PIC), MAF, and observed heterozygosity (Ho) of the population and inferred groups were calculated with the popgen function in the R package “snpReady” (Granato et al., 2018). The analysis of molecular variance (AMOVA) was assessed using the R package “poppr” (Kamvar, Tabima & Grünwald, 2014; Kamvar, Brooks & Grünwald, 2015), and the function ‘randtest’ was performed with 999 replicates for permutation test. Pairwise levels of differentiation and nucleotide diversity (*π*) were estimated using the Populations program in Stacks pipeline (Rochette & Catchen, 2017), and one thousand bootstraps for all loci were set to build 95% confidence intervals for pairwise FST. The R package Core Hunter 3.0 (De Beukelaer, Davenport & Fack, 2018) was employed to develop a core germplasm set with the biparental genomic matrix that contained the total SNPs. The genotypic data of the core germplasm accessions were screened out with VCFtools for further analysis.

## Results

### SLAF-Seq genotyping

A total of 234 taro accessions, including 218 of multi-cormel type and 16 of multi-corm type from 16 provinces of China (Table S1), were surveyed for SNP identification in the present study. Through SLAF-seq, 1774.09 million of clean reads were obtained from this experiment, and number of SLAF tags ranged from 231,112 to 600,960 with an average sequencing depth of 18.04 (Table S2). The average of Q30 reached 93.50% (Table S2) and the average of GC was 45.11% (Table S2) among 234 samples. SNPs were developed for 234 samples based on the polymorphic SLAF tags. After filtering out the invalid SNPs, 132,869 high-quality SNP markers (Table S3) were retained for further analysis.

### Population structure analysis

**Figure 3:**
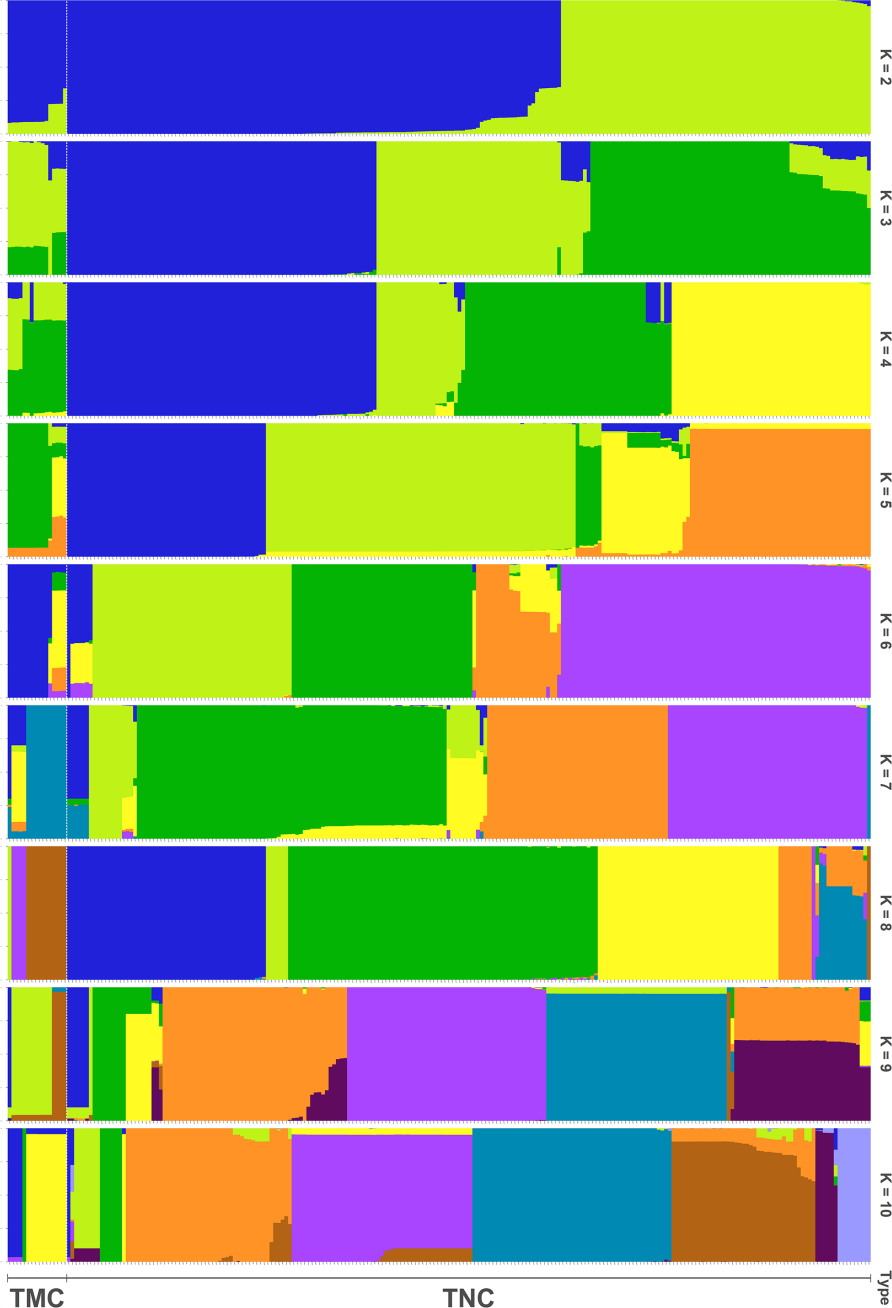
Population structure analysis assuming admixture with 2∼10 clusters. The *x*-axis shows different accessions and the *y*-axis indicates membership probability of accessions belonging to different groups. Horizontal lines at the bottom indicate membership of multi-corm (TMC) and multi-cormel (TNC) type.

**Figure 4:**
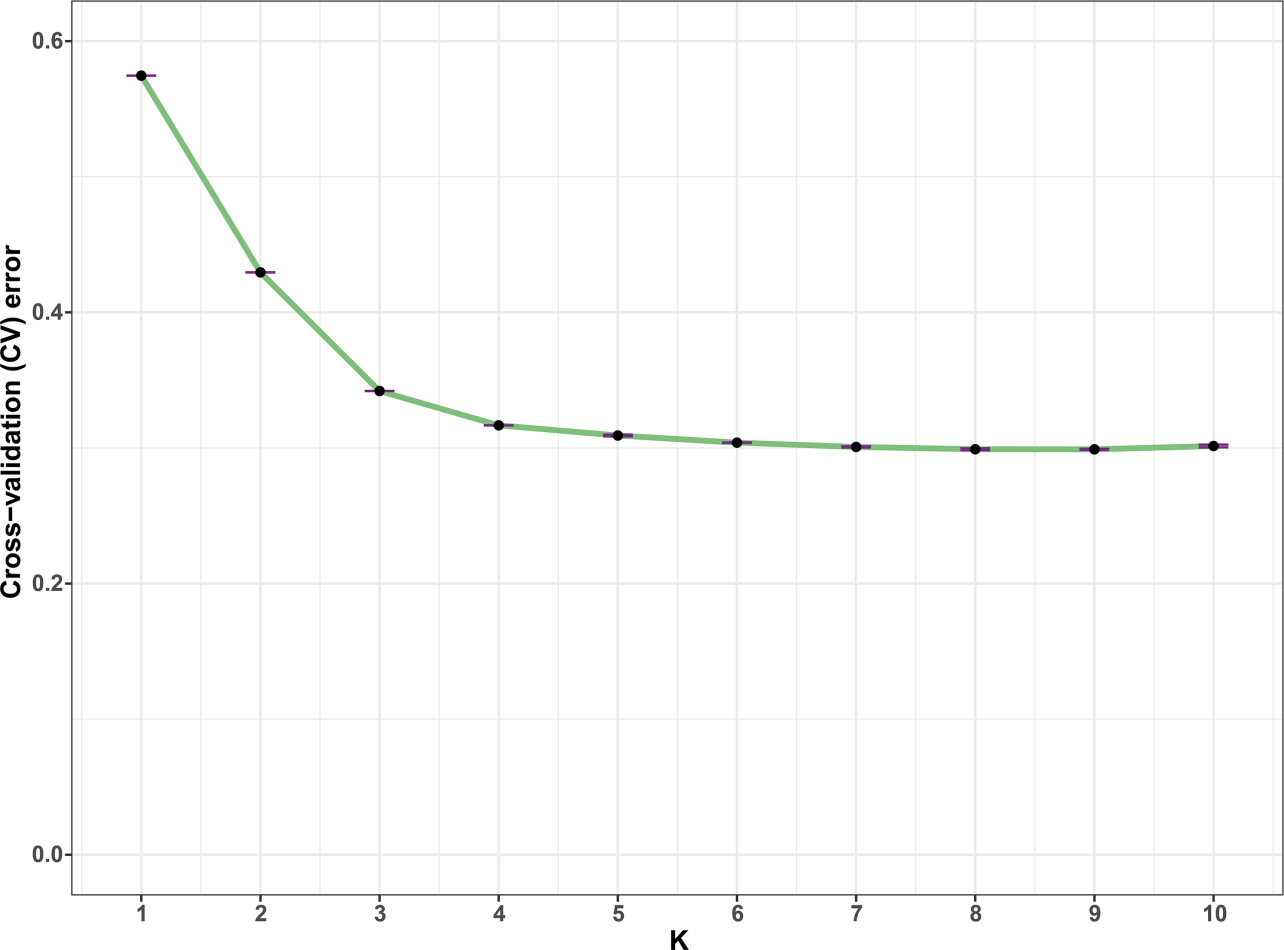
Estimation of cross-validation errors for K values ranging from 1 to 10. The cross-validation errors declined rapidly form *K* = 2 ∼ 5 and reached the lowest value at *K* = 8, which were calculated based on ten independent simulations.

In all, 25505 SNPs passed the filters with HWE of *p* value less than 0.0001 and LD with r^2^ threshold of 0.1. BayeScan detected 13 outlier SNPs which were the subset of the 7,582 ones that identified by Lositan. After screening by these filters, 17,923 SNPs were set to ADMIXTURE for population structure analysis. The estimated ancestry fractions of the 234 accessions for different values of K ranged from 1 to 10 were determine d by ADMIXTURE (Fig. 3). When *K* = 2 ∼ 5, the SNP panel set cannot separate the two morphological types (TMC and TNC) clearly. From *K* = 6 on, the genetic substructure between TMC and TNC progressively takes shape. The cross-validation error (CV) decreased to the lowest value at *K* = 8 (Fig. 4), which indicated that the entire population could be categorized into eight inferred groups. According to membership probability values, six, 12, 54,12, 84, 49, seven and 10 accessions were assigned to group I, II, III, IV, V, VI, VII and VIII, respectively (Fig. 3, Table 1). Accessions in group III, IV, V, VI and VIII are all multi-cormel type, while accessions of multi-corm type dominate in groups I and II, and only one of seven accessions in group VII belongs to the multi-corm type. Grouping of the 234 taro accessions was not according to their collected regions (Table 1).

**Table 1:** Distribution of collected accessions between taro types and inferred groups.

		Group								Total
		I	II	III	IV	V	VI	VII	VIII	
Collection region	Anhui	0	0	1	1	6	2	0	0	10
	Chongqing	0	0	0	0	0	1	0	0	1
	Fujian	3	0	1	0	0	0	0	2	6
	Guangdong	0	0	2	0	3	2	0	2	9
	Guangxi	0	0	4	0	0	0	0	0	4
	Guizhou	0	0	0	0	2	2	0	0	4
	Hainan	0	0	0	0	1	0	0	2	3
	Henan	0	0	0	0	3	0	1	0	4
	Hubei	0	1	2	2	28	5	2	1	41
	Hunan	0	1	0	0	3	1	0	1	6
	Jiangsu	0	0	6	0	6	2	0	0	14
	Jiangxi	1	0	14	0	6	2	0	1	24
	Shandong	0	0	0	1	3	0	2	0	6
	Sichuan	1	9	16	6	17	31	1	0	81
	Yunnan	1	1	1	0	3	0	1	1	8
	Zhejiang	0	0	7	2	3	1	0	0	13
Total		6	12	54	12	84	49	7	10	234
Type	TMC	4	11	0	0	0	0	1	0	16
	TNC	2	1	54	12	84	49	6	10	218
Core set		3	5	5	2	16	7	2	1	41

**Notes.**

TMC the multi-corm type TNC the multi-cormel type Core set number of core germplasm accessions screened by Core Hunter

**Figure 5:**
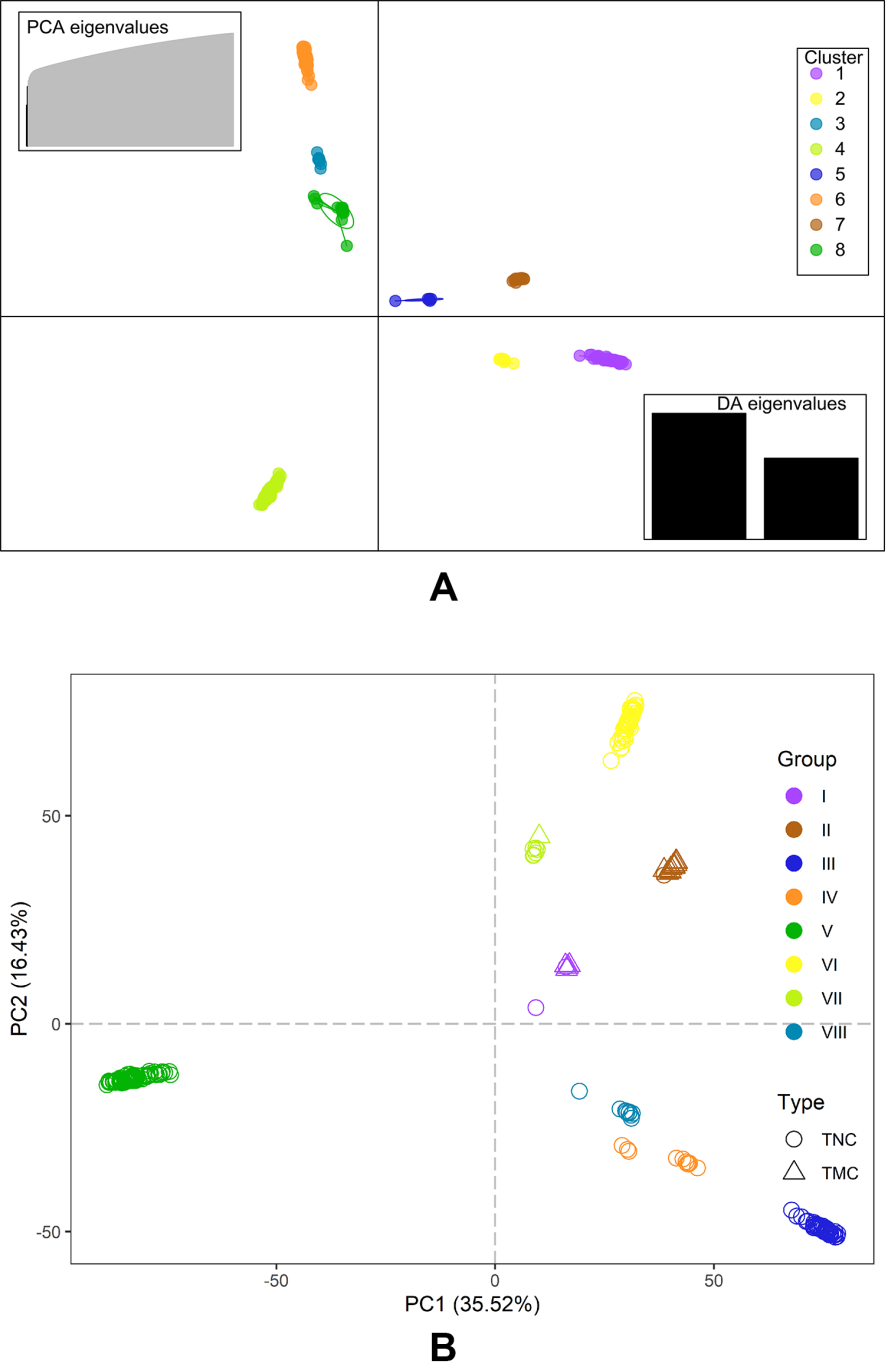
DAPC scatter and PCA plot of the 234 accessions. (A) DAPC scatter plot of the 234 accessions using 40 principal components and 8 discriminant analysis. Circles in the same colors indicate samples in the same clusters that revealed by DAPC. (B) PCA plot of the 234 accessions using the top two principal components based on 132,869 high-quality SNPs . The circles and triangles indicate multi-cormel (TNC) and multi-corm (TMC) type taro, respectively, shapes in different colors represent the inferred groups revealed by ADMIXTURE.

**Figure 6:**
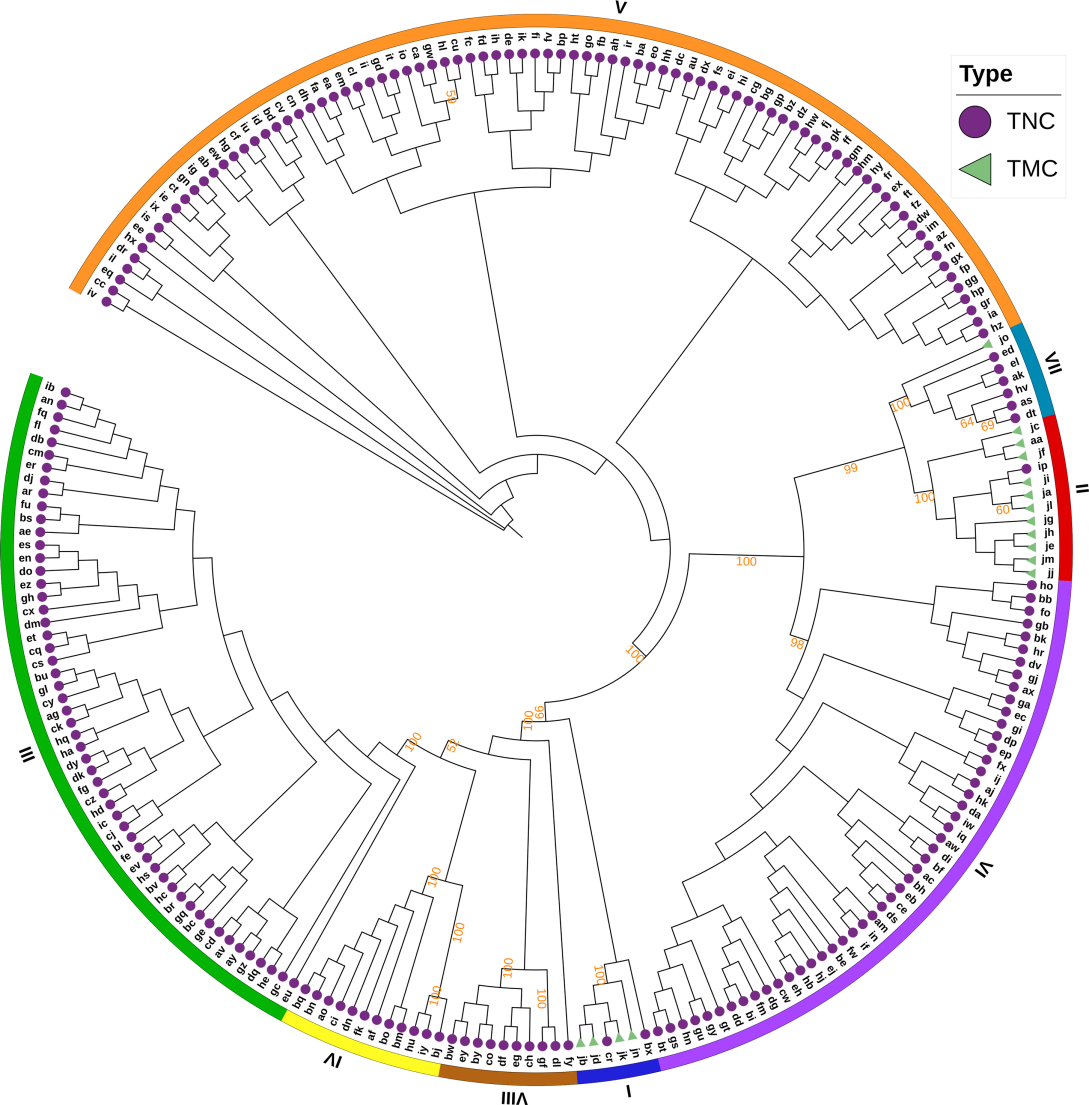
A maximum likelihood phylogenetic tree of the 234 taro accessions based on 132,869 SNPs. The purple circles and green triangles at the end of each branch indicate multi-cormel (TNC) and multi-corm (TMC) type taro of each sample, respectively. The outmost colored strips indicate the inferred eight groups according to the result of ADMIXTURE. Bootstrap values higher than 50% are shown as percentages based on 200 replicates.

The Bayesian information criterion (BIC) from discriminant analysis of principal components (DAPC) was performed to determine the appropriate number of clusters. The BIC showed rapid decline from 1 to 8 (Fig. S2), indicating that the accessions can be grouped into eight main clusters, consistently with the results obtained using ADMIXTURE (Fig. 5A, Fig. S1 and Table S1). To gain deeper insight into the accessions grouping and the pattern of variation, principal component analysis (PCA) was performed to measure the variation in taro collection based on the 132,869 SNP markers. In our study, the first two principal coordinates explained 51.95% of the total variations. It revealed that the eight groups inferred by ADMIXTURE were clearly differentiated in the top two eigenvectors, which accounted for 35.52% and 16.43% of the total variation, respectively (Fig. 5B, Fig. S2). To determine the genetic relationship among taro accessions, a maximum likelihood (ML) phylogenetic tree was constructed based on the SNPs unveiled. As Fig. 6 exhibited, the ML tree can be classified into eight clusters, which showed high consistency with the assignments made by the ADMIXTURE program.

### Genetic diversity analysis

To evaluate the genetic diversity of the eight inferred groups, genetic parameters including nucleotide diversity (*π*), Nei’s genetic diversity index (H), observed heterozigosity, PIC, and MAF were estimated separately. The value of *π* and Nei’s genetic diversity index indicated that accessions in group V exhibited the highest genetic variation, whereas the accessions in group IV and VIII showed the lowest genetic variation (Table 2). Consistently, the highest Nei’s genetic diversity index was present in group V (0.474), and the lowest was found in groups VIII (0.353). Furthermore, The mean PIC values ranged from 0.276 to 0.356, with the highest polymorphism in group V, while the lowest value in group VIII; and mean MAF values revealed similar tendency for the eight groups inferred, which ranged from 0.278 in group VIII to 0.425 in group V and VI.

### Population differentiation

Pairwise *F*_st_ and analysis of molecular variance (AMOVA) was performed to measure genetic differentiation among the eight inferred groups. The results revealed the highest differentiation between group IV and VII, with an *F*_st_ of 0.298. On the other hand, the lowest Fst of 0.100 were found between group VI and II, and group VI and VII, which indicated that they were most closely related (Table 3). The hierarchical AMOVA for all retained SNPs indicated that 77.94% of the total variance was attributed to genetic differentiation among the eight inferred groups, while 22.06% of the diversity was attributed to the genetic differentiation within inferred groups, suggesting a rich genetic diversity level among groups, while 20.46% variance was found among two types and less variance among surveyed 16 regions (11.69%) (Table 4). This is in agreement with the results of population structure, where the inferred groups of taro accessions were not clustered accordingly to their collected regions.

### Development of the core germplasm set

A core germplasm set comprising 41 accessions that included eight multi-corm accessions and 33 multi-cormel accessions, was screened out using Core Hunter (Table 1, Table S1). The genetic diversity of this core germplasm set was estimated to determine the extent to which it might effectively represent the genetic diversity of the entire collection. Comparisons of all genetic parameters revealed that Nei’s genetic diversity index, *π*, Ho, PIC, and MAF values for the core germplasm set were equal to or greater than those for the entire collection (Table 5). Specifically, mean Nei’s genetic diversity, PIC and MAF values of the entire collection were 0.374, 0.299 and 0.279, respectively. However, these values increased to 0.388, 0.307 and 0.295 for the core germplasm set, respectively. Also, the mean *π* and Ho values of the core germplasm set exhibited no significant difference to that of entire collection. The AMOVA result showed that there was no significant difference between the developed core collection and the rest of the collection, and that the ‘within collection’ differences contributed 99.71% of the total genetic variation, indicating that the core collection represented the entire collection well (Table 6).

## Discussion

Taro is important for food security and economics, especially in developing countries and regions. Due to the large genome size and high heterozygosity of taro (Miyasaka et al., 2019), the genetics and genomics research on this species had been relatively neglected. However, progress in transcriptome sequencing and genotyping-by-sequencing (GBS) has allowed genetic analysis of taro through next-generation sequencing strategies. Thus, for example, RNA-seq was performed to identify candidate genes for major metabolic pathways and to develop SSR markers (Liu et al., 2015; You et al., 2015; Wang et al., 2017b). Similarly, Shintaku et al. (2016) developed 240 high-quality SNPs using GBS to construct a genetic map and identify the loci linked to taro leaf-blight resistance. In turn, Soulard, Mournet & Guitton (2017) characterized 22734 and 16744 SNPs to construct two genetic linkage maps with unprecedented chromosome coverage; further, Helmkampf et al. (2017) identified more than 2400 high-quality SNPs to uncover the phylogenetic relationships between taro accessions of different origins. Recently, the whole genome sequence of taro has been published (Bellinger et al., 2020; Yin et al., 2020). The 132,869 genome-wide SNPs unveiled in this study is the largest number of DNA markers for taro to date, which will provide more sequence information, and a powerful tool with high potential for germplasm characterization and molecular breeding of taro.

**Table 2:** Genetic parameters among the eight groups.

Group	H		*π*		Ho		MAF		PIC	
	mean	range	mean	range	mean	range	mean	range	mean	range
I	0.384	0.150∼0.500	0.256	0∼1	0.569	0∼1	0.318	0.080∼0.500	0.302	0.140∼0.380
II	0.462	0.150∼0.500	0.264	0∼1	0.783	0∼1	0.416	0.080∼0.500	0.353	0.140∼0.380
III	0.468	0.100∼0.500	0.197	0∼0.505	0.793	0∼1	0.421	0.060∼0.500	0.354	0.100∼0.380
IV	0.384	0.150∼0.500	0.157	0∼0.667	0.511	0∼1	0.305	0.080∼0.500	0.303	0.140∼0.380
V	0.471	0.100∼0.500	0.320	0∼0.503	0.790	0∼1	0.425	0.050∼0.500	0.356	0.090∼0.380
VI	0.467	0.100∼0.500	0.278	0∼0.506	0.796	0∼1	0.425	0.050∼0.500	0.354	0.090∼0.380
VII	0.434	0.130∼0.500	0.306	0∼1	0.710	0∼1	0.382	0.070∼0.500	0.334	0.120∼0.380
VIII	0.353	0.100∼0.500	0.187	0∼1	0.463	0∼1	0.278	0.050∼0.500	0.276	0.090∼0.380

**Notes.**

H Nei’s genetic diversity index PIC polymorphic information content MAF minor allelefrequency *π* nucleotide diversity Ho observed heterozygosity

Range indicates minimal to maximum value.

**Table 3:** Matrix of pairwise Nei’s genetic distance and *F*_*st*_ among the eight inferred groups.

Inferred group	I	II	III	IV	V	VI	VII	VIII
I	0	0.209	0.215	0.176	0.251	0.166	0.246	0.147
II	0.218	0	0.165	0.220	0.308	0.099	0.100	0.219
III	0.203	0.187	0	0.144	0.345	0.206	0.218	0.146
IV	0.230	0.258	0.155	0	0.315	0.246	0.276	0.098
V	0.138	0.199	0.278	0.191	0	0.277	0.232	0.265
VI	0.132	0.100	0.213	0.204	0.220	0	0.102	0.204
VII	0.244	0.135	0.220	0.298	0.143	0.100	0	0.232
VIII	0.193	0.244	0.154	0.155	0.157	0.168	0.255	0

**Notes.**

Above diagonal Nei’s genetic distance; below diagonal: Fst.

**Table 4:** Analysis of molecular variance (AMOVA) of the entire taro accessions.

Source	DF	SS	MS	Components of covariance	
				Sigma	(%)^a^
*Sixteen provinces of collected regions*					
Between	15	133075.4	8871.69	434.5	11.69
Within	218	715281.2	3281.11	3281.11	88.31
Total	233	848356.6	3641.02	3715.61	100
*Two morphological-types*					
Between	1	30549.93	30549.93	906.51	20.46
Within	232	817806.7	3525.03	3525.03	79.54
Total	233	848356.6	3641.02	4431.54	100
*Eight groups based on ADMIXTURE and DAPC*					
Between	7	626898.1	89556.87	3461.92	77.94
Within	226	221458.5	979.9	979.9	22.06
Total	233	848356.6	3641.02	4441.83	100

**Notes.**

DF degree of freedom SS sum of square MS mean of square

a These variances were statistically significant from zero at *P* < 0.001.

**Table 5:** Comparison of genetic diversity indices between the entire collection and coregermplasm set.

Collection	H		*π*		Ho		PIC		MAF	
	mean	range	mean	range	mean	range	mean	range	mean	range
Entire collection	0.374	0.170∼0.500	0.375	0.180∼0.500	0.438	0∼1	0.299	0.160∼0.380	0.279	0.050∼0.500
Core germplasm set	0.388^**^	0.180∼0.500	0.375	0.180∼0.500	0.438	0∼1	0.307^**^	0.160∼0.380	0.295^**^	0.050∼0.500

**Notes.**

H Nei’s genetic diversity index PIC polymorphic information content MAF minor allele frequency *π* nucleotide diversity Ho observed heterozygosity

** indicates significant difference in genetic diversity parameter at 0.01 level between the entire and core germplasm set collection revealed by *t*-test.

**Table 6:** Result of the AMOVA between the core collection and the rest of the entire collection.

Source of variation	DF	SS	MS	Components of covariance		*P*-value
				Sigma	%	
Between collection	1	4358.87	4358.87	10.66	0.29	0.259
Within collection	232	843997.73	3637.92	3637.92	99.71	
Total	233	848356.60	3641.02	3648.58	100	

**Notes.**

DF degree of freedom SS sum of square MS mean of square

Huang et al. (2016) divided the multi-corm and multi-cormel taro types into two and three subtypes, according to petiole and bud color, respectively. In this study, the joint analyses of population structure (Fig. 3), phylogenetic relationships (Fig. 6), and PCA (Fig. 5B), revealed that the total of surveyed taro accessions could be divided into eight groups. Most accessions of the multi-corm type were divided into two groups, similar to the study reported by Huang et al. (2016). While the multi-cormel accessions were categorized into at least six groups, which is a larger number of groups than that obtained on the basis of morphological characterization, most likely because of a more precise genotyping within continuous morphological variation in our study.

Although all accessions in five of the eight groups are of the multi-cormel type taro, the phylogenetic tree (Fig. 5) based on SNPs failed to separate the multi-corm and multi-cormel genotypes completely. The two types showed very low genetic differentiation (with a pairwise *F*_st_ value of 0.019). We speculate that the multi-corm type evolved from the multi-cormel taro type during the process of domestication and evolution (Xu et al., 2001), which might lead to the high phylogenetic similarity among a few accessions of the two types.

Taro accessions with the same geographical origin did not cluster into the same groups in this study (Table 1, Table S1), and AMOVA revealed that only 11.69% (Table 4) of the genetic diversity was attributed to the genetic differentiation among the 16 surveyed regions. As taro is propagated asexually, introduction breeding among different regions has occurred frequently, possibly promoting exchange of genetic material and resulting in similar genetic background among regions (Table 4). Hu et al. (2015) found that 110 accessions of taro were grouped into six clusters based on 10 SSR markers and varieties with the same or similar morphological type are genetically related except of a little divergence, while the geographical origin had less effect on the genetic variation. In sweet potato, Su et al. (2017) reported that the genetic distances between geographic regions were low; Wadl et al. (2018) also found instances of accessions clustering into groups that did not match the geographic location. Similar studies in potato also revealed a lack of geographical differentiation among country-wide collections (Wang et al., 2017a). These reports suggested that germplasm introduction of clonal crops was common among regions.

The conservation and utilization of taro germplasm are the main aims of the National Germplasm Repository for Aquatic Vegetables. In this study, a core germplasm set including 41 taro accessions was identified based on our genome-wide discovery of 132,869 SNPs. Furthermore, this core germplasm set is highly representative of the genetic diversity of the entire collection (Table 5). Taro is an asexually propagated crop with abundant germplasm resources whose management is labor intensive. The core germplasm set was selected with a relatively low sampling intensity (17.95% of the entire collection) which, nonetheless, proved highly efficient in maintaining genetic diversity of the entire collection. This will allow manual labor involved in genetic conservation of taro to be substantially reduced.

This report is the first attempt to develop core germplasm of taro in China, which will prevent redundant introduction breeding in taro and assist breeders to use the accessions effectively. Additionally, through eliminating genetically similar germplasm, we can focus on suitable agronomic traits with a relatively small number of taro accessions that could be used as parents for further breeding programs.

## Conclusions

Through SLAF-seq, 132,869 high-quality SNPs were identified in this study. This is the largest number of SNP markers developed for taro to the present. These SNP markers were effectively used in the evaluation of taro genetic diversity and population differentiation. The results of population structure, phylogenetic relationships and principal component analysis (PCA) based on the SNPs revealed that the 234 taro accessions of China can be classified into eight groups. The majority of total variance was attributed to genetic differentiation among the eight inferred groups but not to the collected regions. Furthermore, a core germplasm set comprising 41 taro accessions were identified which highly maintained the overall of genetic diversity of entire collection to a great extent. In addition to significantly expanding the genomics and genetics information on taro, these findings will prove highly valuable for genetic characterization, germplasm resources conservation and breeding of taro.

##  Supplemental Information

10.7717/peerj.10485/supp-1 Supplemental Information 1 Graph of Bayesian Information Criterion versus number of clusters

10.7717/peerj.10485/supp-2 Supplemental Information 2 Relative eigenvalues of the first 10 principal components

10.7717/peerj.10485/supp-3 Supplemental Information 3 Detail information of the 234 taro accessions collected in this study TMC: the multi-corm type, TNC: the multi-cormel type; Inferred group: group revealed by ADMIXTURE; NA: not accessible. T indicates accession belonging to the core germplasm.

10.7717/peerj.10485/supp-4 Supplemental Information 4 Sequencing and identified SNPs statistics of 234 taro accessions

10.7717/peerj.10485/supp-5 Supplemental Information 5 List of the 132869 SNPs.

## Additional Information and Declarations

### Data Availability

The following information was supplied regarding data availability: The sequences are available at the SRA: PRJNA644023.
