# Supplementary figures and images for: Genetic diversity and population structure of eddoe taro in China using genome-wide SNP markers
Source: PeerJ. 2020 Dec 8;8:e10485. doi: 10.7717/peerj.10485 (PMC7731653; doi:10.7717/peerj.10485)

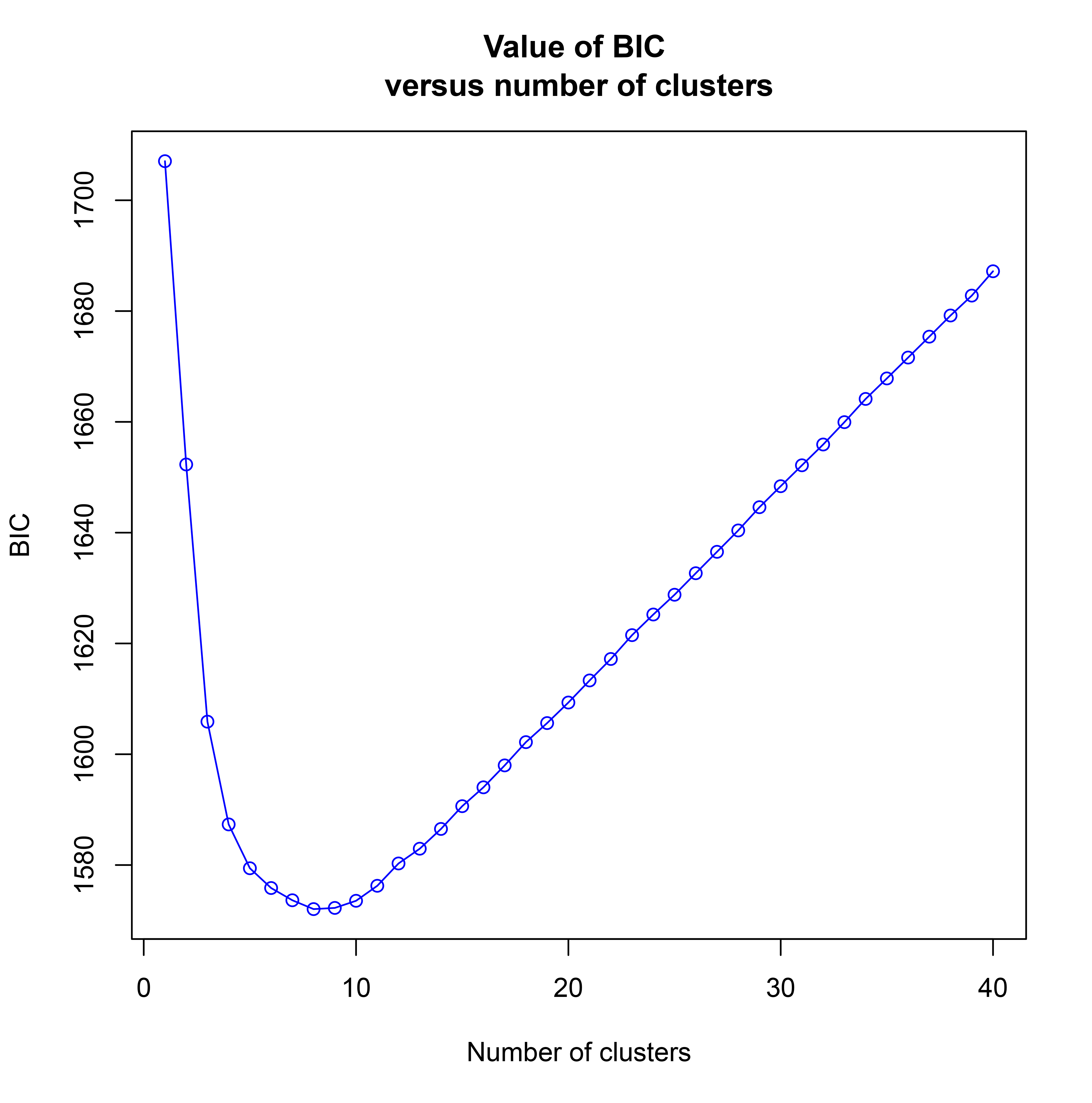

Supplement: Supplemental Information 1 [file peerj-08-10485-s001.jpg]

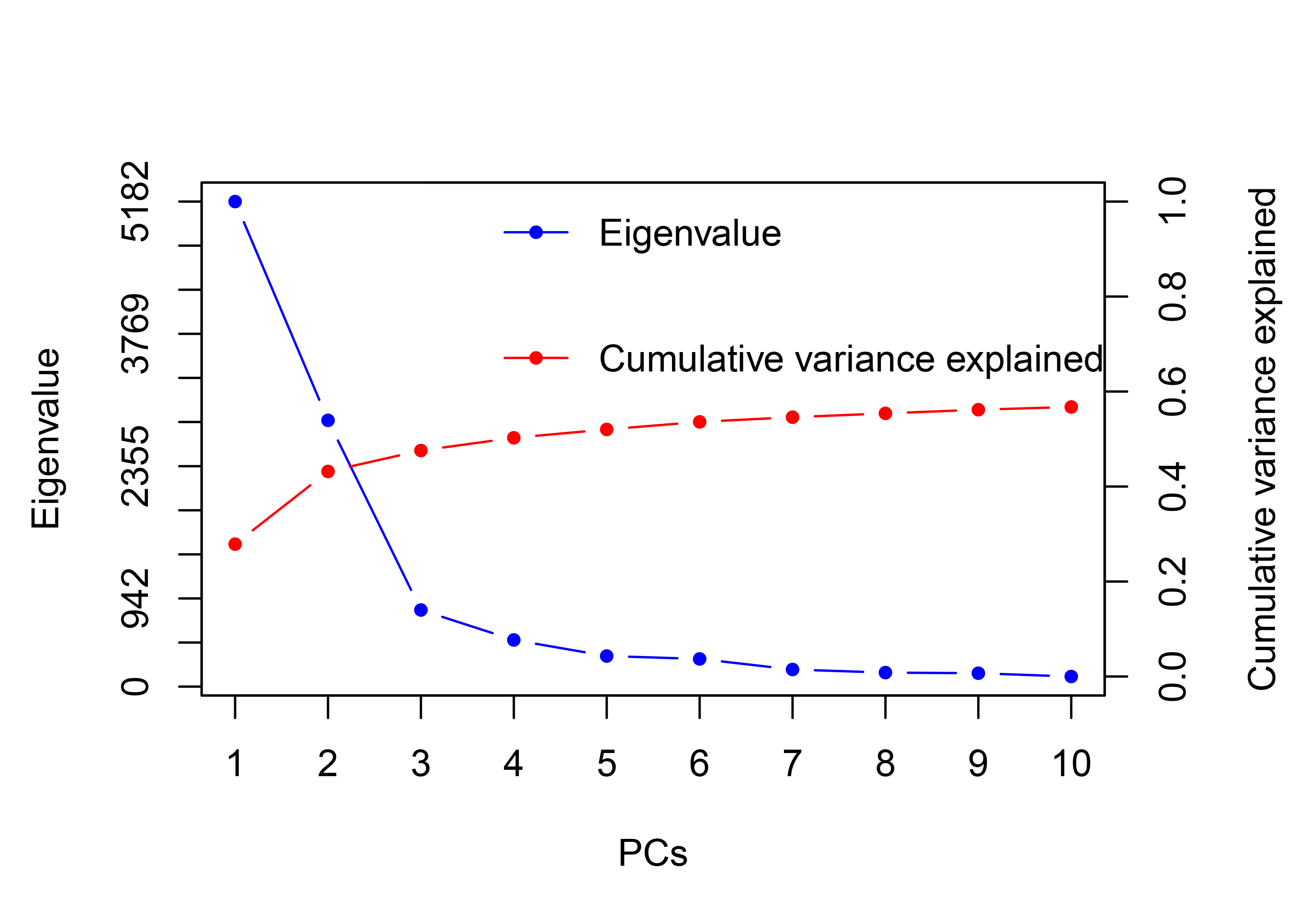

Supplement: Supplemental Information 2 [file peerj-08-10485-s002.jpg]
