# Supplementary material for: Genetic diversity and population structure of eddoe taro in China using genome-wide SNP markers
Source: PeerJ. 2020 Dec 8;8:e10485. doi: 10.7717/peerj.10485 (PMC7731653; doi:10.7717/peerj.10485)
Supplement: Supplemental Information 3 — TMC: the multi-corm type, TNC: the multi-cormel type; Inferred group: group revealed by ADMIXTURE; NA: not accessible. T indicates accession belonging to the core germplasm. [file peerj-08-10485-s003.docx]

**Table S1. Detail information of the 234 taro accessions collected in this study.**

| No | Sample code | Variety | Collection Province | Collection City/County | Type | Inferred group | DAPC cluster |
| --- | --- | --- | --- | --- | --- | --- | --- |
| 1 | bx | Yulinhonggeng | Yunnan | Mengla | TNC | I | 1 |
| 2 | cr | Dongbaoyu | Jiangxi | Linchuan | TNC | I | 1 |
| 3 | jb | Goutiyu | Fujian | Zhangzhou | TMC | I | 1 |
| 4 | jd | Cannaduotou | Fujian | Anxi | TMC | I | 1 |
| 5 | jk | Jiuzhangyu | Fujian | NA | TMC | I | 1 |
| 6 | jn | Zhugenyu | Sichuan | Anxi | TMC | I | 1 |
| 7 | aa | Changshajipoyu | Hunan | Changsha | TMC | II | 7 |
| 8 | ip | 9896 | Yunnan | Kunming | TNC | II | 7 |
| 9 | ja | Mianyangduotouyu | Sichuan | Mianyang | TMC | II | 7 |
| 10 | jc | Zizhonglianhuayu | Sichuan | Zizhong | TMC | II | 7 |
| 11 | je | Guanghuaduotouyu | Hubei | Qianjiang | TMC | II | 7 |
| 12 | jf | Xuanhangoutouyu | Sichuan | Dazhou | TMC | II | 7 |
| 13 | jg | Jiangeedanyu | Sichuan | Guangyuan | TMC | II | 7 |
| 14 | jh | Ziyangjiangbayu | Sichuan | Ziyang | TMC | II | 7 |
| 15 | ji | Doujiangyanyutangyu | Sichuan | Dujiangyan | TMC | II | 7 |
| 16 | jj | Jianyangjipoyu | Sichuan | Jianyang | TMC | II | 7 |
| 17 | jl | Renshougedayu | Sichuan | Renshou | TMC | II | 7 |
| 18 | jm | Yibinlianhuayu | Sichuan | Yibin | TMC | II | 7 |
| 19 | ae | Quxianmaoyuzi | Sichuan | Quxian | TNC | III | 5 |
| 20 | ag | Hongganyu | Guangdong | NA | TNC | III | 5 |
| 21 | an | Xuanhanhanyu | Sichuan | Xuanhan | TNC | III | 5 |
| 22 | ar | Xuanbaoyu | Jiangsu | Taixing | TNC | III | 5 |
| 23 | av | Penghuaganyuzi | Sichuan | Pengan | TNC | III | 5 |
| 24 | ay | Guangchanghongyayu | Jiangxi | Guangchang | TNC | III | 5 |
| 25 | bc | Tangjiangyu-3 | Jiangxi | Nankang | TNC | III | 5 |
| 26 | bl | Dayihanyu | Sichuan | Dayi | TNC | III | 5 |
| 27 | br | Renshoushuiyu | Sichuan | Renshou | TNC | III | 5 |
| 28 | bs | Dongshanhongzuiyu | Sichuan | Ziyang | TNC | III | 5 |
| 29 | bu | Chongzhouhongzuiyu | Sichuan | Chongzhou | TNC | III | 5 |
| 30 | bv | Jiantianyuyutou | Jiangxi | Jian | TNC | III | 5 |
| 31 | cd | Huzhouhongheyu | Zhejiang | Huzhou | TNC | III | 5 |
| 32 | cj | Guiganghongyayu | Guangxi | Guigang | TNC | III | 5 |
| 33 | ck | Danxiahongyayu | Guangdong | Renhuaxian | TNC | III | 5 |
| 34 | cm | Tongchuanyuzi | Sichuan | Tongchuan | TNC | III | 5 |
| 35 | cq | Qianshanhongyayu | Jiangxi | Yanshan | TNC | III | 5 |
| 36 | cs | Zizhonghongheyu | Sichuan | Zizhong | TNC | III | 5 |
| 37 | cx | Linchuanqinghehongyahanyu-1 | Jiangxi | Linchuan | TNC | III | 5 |
| 38 | cy | Ruijinyu | Jiangxi | Ruijin | TNC | III | 5 |
| 39 | cz | Wuyu-1 | Hubei | Wuhan | TNC | III | 5 |
| 40 | db | Xuedousiyu | Zhejiang | Fenghua | TNC | III | 5 |
| 41 | dj | Shetanhanyu | Sichuan | Fengdu | TNC | III | 5 |
| 42 | dk | Jintanghongzuiyu | Sichuan | Cangxi | TNC | III | 5 |
| 43 | dm | Anfuhongyayu | Jiangxi | Anfu | TNC | III | 5 |
| 44 | do | Qingyanghongxinyu | Anhui | Qingyang | TNC | III | 5 |
| 45 | dq | Longyouhongyayu | Zhejiang | Longyou | TNC | III | 5 |
| 46 | dy | Yingtanhongyayu | Jiangxi | Yingtan | TNC | III | 5 |
| 47 | en | Pujihongyu | Hubei | Zhoushan | TNC | III | 5 |
| 48 | er | Wuzhouyu | Guangxi | Wuzhou | TNC | III | 5 |
| 49 | es | Ningbodaxiehongyutou | Zhejiang | Ningbo | TNC | III | 5 |
| 50 | et | Hongyanyu | Guangxi | Wuzhou | TNC | III | 5 |
| 51 | eu | Huangyanhongyaduoziyu | Zhejiang | Huangyan | TNC | III | 5 |
| 52 | ev | Denglongfenyu | Sichuan | Jian | TNC | III | 5 |
| 53 | ez | Dongxianghongyayu | Jiangxi | Dongxiang | TNC | III | 5 |
| 54 | fe | Taixingxiangheyu | Jiangsu | Taizhou | TNC | III | 5 |
| 55 | fg | Wuyishanhongyayu | Fujian | Wuyishan | TNC | III | 5 |
| 56 | fl | Xiangshayu | Jiangsu | Taizhou | TNC | III | 5 |
| 57 | fq | Hongyalianhuayu | Sichuan | Huangshan | TNC | III | 5 |
| 58 | fu | Lepinghongyayu | Jiangxi | Leping | TNC | III | 5 |
| 59 | gc | Yiqianduoziyu | Jiangxi | Guangchang | TNC | III | 5 |
| 60 | ge | Ziyanghongganyu-1 | Sichuan | Ziyang | TNC | III | 5 |
| 61 | gh | Yiqianyu | Jiangxi | Guangchang | TNC | III | 5 |
| 62 | gl | Liyanghongyayu | Jiangsu | Liyang | TNC | III | 5 |
| 63 | gq | Boyanghanyu | Jiangxi | Boyang | TNC | III | 5 |
| 64 | gz | Yiliangqingganyu | Yunnan | Yiliang | TNC | III | 5 |
| 65 | ha | Emeishanbaiganhongyayu | Sichuan | Emeishan | TNC | III | 5 |
| 66 | hc | Wuchenghongyu | Zhejiang | Jinhua | TNC | III | 5 |
| 67 | hd | Nanninghongyayu-1 | Guangxi | Nanning | TNC | III | 5 |
| 68 | he | Huangqiaozhenyu | Jiangsu | Taixing | TNC | III | 5 |
| 69 | hq | Zhejianghongyayu | Zhejiang | NA | TNC | III | 5 |
| 70 | hs | Doujiangyanhongyayu-2 | Sichuan | Dujiangyan | TNC | III | 5 |
| 71 | ib | Jinggangshanyutou | Jiangxi | Jinggangshan | TNC | III | 5 |
| 72 | ic | Nanjingliuheyu | Jiangsu | Nanjing | TNC | III | 5 |
| 73 | af | Hanyanghonghe | Hubei | Wuhan | TNC | IV | 6 |
| 74 | ao | Dongshanheiganyu | Sichuan | Dongshan | TNC | IV | 6 |
| 75 | bj | Fenghuadayunai | Zhejiang | Fenghua | TNC | IV | 6 |
| 76 | bm | Xiaziyu | Shandong | Laiyang | TNC | IV | 6 |
| 77 | bn | Xuehuhongheyu | Anhui | Qianshan | TNC | IV | 6 |
| 78 | bo | Pengshanheiganyu | Sichuan | Pengshan | TNC | IV | 6 |
| 79 | bq | Jianyanghongyu | Sichuan | Jianyang | TNC | IV | 6 |
| 80 | ci | Daxianhongyu | Sichuan | Daxian | TNC | IV | 6 |
| 81 | dn | Jianbayu | Sichuan | NA | TNC | IV | 6 |
| 82 | fk | Huangchenghonggengyu-2 | Sichuan | Xianfeng | TNC | IV | 6 |
| 83 | hu | Huachenghongyayu | Zhejiang | Ningbo | TNC | IV | 6 |
| 84 | iy | Huangtanzhanyu | Hubei | Yingcheng | TNC | IV | 6 |
| 85 | ab | Yanbianjidanyu | Sichuan | Yanbian | TNC | V | 8 |
| 86 | ah | Jinanyu | Shandong | Jinan | TNC | V | 8 |
| 87 | au | Baigenhanxiangyu | Sichuan | Daxian | TNC | V | 8 |
| 88 | az | Hongshanliqiaoyu | Hubei | Wuhan | TNC | V | 8 |
| 89 | ba | Nanyangyu | Henan | Nanyang | TNC | V | 8 |
| 90 | bd | Hangzhouyu | Zhejiang | Hangzhou | TNC | V | 8 |
| 91 | bg | Shenjiamenyunai | Zhejiang | Zhoushan | TNC | V | 8 |
| 92 | bp | Laifengbaihe | Hubei | Laifeng | TNC | V | 8 |
| 93 | bz | Sanlvduoziyu | Hunan | Changde | TNC | V | 8 |
| 94 | ca | Dayeyu-1 | Hubei | Daye | TNC | V | 8 |
| 95 | cc | Huangguoshuqingganyu | Guizhou | Anshun | TNC | V | 8 |
| 96 | cf | Wangyingbaihe | Hubei | Lichuan | TNC | V | 8 |
| 97 | cg | Guangdongduoziyu | Guangdong | Guangzhou | TNC | V | 8 |
| 98 | cl | Yingshanyuzi | Sichuan | Yingshan | TNC | V | 8 |
| 99 | cn | Daxianbaiyutou | Sichuan | Daxian | TNC | V | 8 |
| 100 | ct | Huangwanduoziyu | Hubei | Qianjiang | TNC | V | 8 |
| 101 | cu | Shandongyu-2 | Shandong | Jinan | TNC | V | 8 |
| 102 | cv | Gaomiaobaiganyu | Hubei | Wuhan | TNC | V | 8 |
| 103 | dc | Jianghanmaoyu | Hubei | Qianjiang | TNC | V | 8 |
| 104 | de | Yichengyeshengshuiyu | Anhui | Xuancheng | TNC | V | 8 |
| 105 | dh | Chongzhouheiganyu | Sichuan | Chongzhou | TNC | V | 8 |
| 106 | dr | Anfubaiyayu | Jiangxi | Anfu | TNC | V | 8 |
| 107 | dw | Baoyingbaiyayu | Jiangsu | Baoying | TNC | V | 8 |
| 108 | dx | Longquanlvgengyeyu | Guizhou | Zunyishi | TNC | V | 8 |
| 109 | dz | Linchuanbaiheyu | Jiangxi | Linchuan | TNC | V | 8 |
| 110 | ea | Zhiwuyuanlvgeng | Yunnan | Wuhan | TNC | V | 8 |
| 111 | ee | Bazhongbaiyu | Shandong | Bazhong | TNC | V | 8 |
| 112 | ei | Shiqiaoshuiyu | Sichuan | Chongqing | TNC | V | 8 |
| 113 | em | Tanglidianyu | Henan | Nanyang | TNC | V | 8 |
| 114 | eo | Shehonglvgengyu | Sichuan | Shehong | TNC | V | 8 |
| 115 | eq | Huzhouyu | Zhejiang | Huzhou | TNC | V | 8 |
| 116 | ew | Dongxiangbaihehuayu | Jiangxi | Dongxiang | TNC | V | 8 |
| 117 | ex | Maweiyu | Anhui | Qingyang | TNC | V | 8 |
| 118 | fa | Dabaobaigeng | Sichuan | Fengdu | TNC | V | 8 |
| 119 | fb | Jiangxiayutou | Hubei | Wuhan | TNC | V | 8 |
| 120 | fc | Gufulvgengyu | Hubei | Yichang | TNC | V | 8 |
| 121 | fd | Yangyu1hao | Jiangsu | Yangzhou | TNC | V | 8 |
| 122 | ff | Peixianyu | Jiangsu | Peixian | TNC | V | 8 |
| 123 | fi | Tianmenlvgengyu | Hubei | Tianmen | TNC | V | 8 |
| 124 | fj | Shiqiaoshehonglvgengyu | Sichuan | Shehong | TNC | V | 8 |
| 125 | fn | Jinkouyutou | Hubei | Wuhan | TNC | V | 8 |
| 126 | fp | Hongxianuoyu | Hubei | Wuhan | TNC | V | 8 |
| 127 | fr | Hunanbaiheyu | Hunan | Changsha | TNC | V | 8 |
| 128 | fs | Hefeiyu | Anhui | Hefei | TNC | V | 8 |
| 129 | ft | Jieyangduoziyu | Guangdong | Jieyang | TNC | V | 8 |
| 130 | fv | Chikanyu | Guangdong | Zhanjiang | TNC | V | 8 |
| 131 | fz | Hejiapingduoziyu | Sichuan | Youxian | TNC | V | 8 |
| 132 | gd | Puxingyuzi | Sichuan | Chengdu | TNC | V | 8 |
| 133 | gg | Xiulinhanyu | Hubei | Shishou | TNC | V | 8 |
| 134 | gk | Wuyicunyu | Anhui | Dangtu | TNC | V | 8 |
| 135 | gm | Xiaogangyu-1 | Hubei | Honghu | TNC | V | 8 |
| 136 | gn | Shetanyu | Sichuan | Fengdu | TNC | V | 8 |
| 137 | go | Rugaoyu | Jiangsu | Rugao | TNC | V | 8 |
| 138 | gp | Jinzibabaigeng | Hubei | Enshi | TNC | V | 8 |
| 139 | gr | Jinzhuyu | Hubei | Zigui | TNC | V | 8 |
| 140 | gw | Shanghubaihehanyu | Hubei | Hanchuan | TNC | V | 8 |
| 141 | gx | Zhangshubaiyanuoyu | Jiangxi | Zhangshu | TNC | V | 8 |
| 142 | hg | Wuyangyu | Henan | Wuyang | TNC | V | 8 |
| 143 | hh | Huangnibagaogeng | Hubei | Enshi | TNC | V | 8 |
| 144 | hi | Huangshiyu | Hubei | Huangshi | TNC | V | 8 |
| 145 | hl | Jingmenyu | Hubei | Jingmen | TNC | V | 8 |
| 146 | hm | Dongcunyu | Anhui | Qingyang | TNC | V | 8 |
| 147 | hp | Qizhouyu | Hubei | Qichun | TNC | V | 8 |
| 148 | ht | Mashanbaiyu-1 | Jiangsu | Wuxi | TNC | V | 8 |
| 149 | hw | Guanghuayu-2 | Hubei | Qianjiang | TNC | V | 8 |
| 150 | hx | Suzhoubaihe | Jiangsu | Suzhou | TNC | V | 8 |
| 151 | hy | Xiangnanjingyu | Hunan | Xiangtan | TNC | V | 8 |
| 152 | hz | Dayehuyu | Hubei | Daye | TNC | V | 8 |
| 153 | ia | Zuoliduoziyu | Jiangxi | Huangshan | TNC | V | 8 |
| 154 | id | Xuanchengyeshengshuiyu | Anhui | Xuanchuan | TNC | V | 8 |
| 155 | ie | Jianmaoziyu | Jiangxi | Jian | TNC | V | 8 |
| 156 | ig | Jiangeliuhelvyu | Sichuan | Jiange | TNC | V | 8 |
| 157 | ih | Hanyanggaomiaobaizhong | Hubei | Wuhan | TNC | V | 8 |
| 158 | ii | Pujibaiheyu | Hubei | Zhoushan | TNC | V | 8 |
| 159 | ik | Hanchuanyu-1 | Hubei | Hanchuan | TNC | V | 8 |
| 160 | il | Suizhouyu | Hubei | Suizhou | TNC | V | 8 |
| 161 | im | Haikoubaihe | Hainan | Haikou | TNC | V | 8 |
| 162 | io | Puzheheilvgengyu | Yunnan | Puzhehei | TNC | V | 8 |
| 163 | ir | Jianshuiyu-1 | Yunnan | Jianshui | TNC | V | 8 |
| 164 | is | Fasibaihe | Hubei | Wuhan | TNC | V | 8 |
| 165 | it | Chuanshanwuganyu | Sichuan | Suining | TNC | V | 8 |
| 166 | iu | Qililvyu | Sichuan | Sichuan | TNC | V | 8 |
| 167 | iv | Baopingkoulvgengyu | Sichuan | Dujiangyan | TNC | V | 8 |
| 168 | ix | Guanganganyuzi | Sichuan | Guangan | TNC | V | 8 |
| 169 | ac | Shehonghongyu | Sichuan | Shehong | TNC | VI | 2 |
| 170 | aj | Penganbaiyu | Sichuan | Pengan | TNC | VI | 2 |
| 171 | am | Tongshanzigengyu | Hubei | Tongshan | TNC | VI | 2 |
| 172 | aw | Chongzhouyuzi | Sichuan | Chongzhou | TNC | VI | 2 |
| 173 | ax | Wufuziganyu | Sichuan | Wuyishan | TNC | VI | 2 |
| 174 | bb | Nanquanbaihehanyu | Chongqing | Chongqing | TNC | VI | 2 |
| 175 | be | Yibinwuganqiang | Sichuan | Yibin | TNC | VI | 2 |
| 176 | bf | Enshihonghe | Hubei | Enshi | TNC | VI | 2 |
| 177 | bh | Shashinuoyu | Hubei | Shashi | TNC | VI | 2 |
| 178 | bi | Guiyangyutou | Guizhou | Guiyang | TNC | VI | 2 |
| 179 | bk | Wangshisanyu-1 | Jiangxi | Fengcheng | TNC | VI | 2 |
| 180 | bt | Qiongxiaheiganyu | Sichuan | Qiongxia | TNC | VI | 2 |
| 181 | ce | Zhaojiyu | Jiangsu | Huaian | TNC | VI | 2 |
| 182 | cw | Langzhonghongganyu | Sichuan | Langzhong | TNC | VI | 2 |
| 183 | da | Huangjinbawuhe | Hubei | Enshi | TNC | VI | 2 |
| 184 | dd | Jixishuiyu | Anhui | Jixi | TNC | VI | 2 |
| 185 | dg | Zizhongheiganyu | Sichuan | Zizhong | TNC | VI | 2 |
| 186 | di | Suishanyutou | Sichuan | Emeishan | TNC | VI | 2 |
| 187 | dp | Luzhouwuganqiangyu | Sichuan | Luzhou | TNC | VI | 2 |
| 188 | ds | Qianfoziyu | Sichuan | Guangyuan | TNC | VI | 2 |
| 189 | dv | Dazhoushuiyu | Sichuan | Dazhou | TNC | VI | 2 |
| 190 | eb | Tangziyu | Sichuan | NA | TNC | VI | 2 |
| 191 | ec | Renshouwuganqiangyu | Sichuan | Renshou | TNC | VI | 2 |
| 192 | eh | Caotanghanyu | Sichuan | Chengdu | TNC | VI | 2 |
| 193 | ej | Yujiacunyu | Jiangxi | Jian | TNC | VI | 2 |
| 194 | ep | Renhezhenwuheyu | Sichuan | Chongqing | TNC | VI | 2 |
| 195 | fm | Jiangyanziganyu | Jiangsu | Taizhou | TNC | VI | 2 |
| 196 | fo | Najianghongganduoziyu | Sichuan | Neijiang | TNC | VI | 2 |
| 197 | fw | Wuganqiang | Sichuan | Luzhou | TNC | VI | 2 |
| 198 | fx | Zhuanheheyu | Sichuan | Yuechi | TNC | VI | 2 |
| 199 | ga | Pingchangshuiyu | Sichuan | Pingchang | TNC | VI | 2 |
| 200 | gb | Guanganshuiyu | Sichuan | Guangan | TNC | VI | 2 |
| 201 | gi | Qianshanwujiaoyu | Anhui | Qianshan | TNC | VI | 2 |
| 202 | gj | Bijieyutou | Guizhou | Bijie | TNC | VI | 2 |
| 203 | gs | Jianyangshiqiaohonggengyu | Sichuan | Jianyang | TNC | VI | 2 |
| 204 | gt | Huachengwuheyu | Zhejiang | Ningbo | TNC | VI | 2 |
| 205 | gu | Yongzhouhonggenghanyu | Hunan | Yongzhou | TNC | VI | 2 |
| 206 | gy | Wujiashanyu | Guangdong | Guangzhou | TNC | VI | 2 |
| 207 | hb | Zigonghongheshuiyu | Sichuan | Zigong | TNC | VI | 2 |
| 208 | hj | Jiangewuganyu | Sichuan | Jiange | TNC | VI | 2 |
| 209 | hk | Mabayu | Guangdong | Shaoguan | TNC | VI | 2 |
| 210 | hn | Laifenghonggeng | Hubei | Enshi | TNC | VI | 2 |
| 211 | ho | Hanzhongziyu | Sichuan | Hanzhong | TNC | VI | 2 |
| 212 | hr | Gayuncunhongganyu | Sichuan | Daxian | TNC | VI | 2 |
| 213 | if | Emeishanziganyu-1 | Sichuan | Emeishan | TNC | VI | 2 |
| 214 | ij | Santaiyuzi | Sichuan | Santai | TNC | VI | 2 |
| 215 | in | Wanguhonggengyu | Sichuan | Mingshan | TNC | VI | 2 |
| 216 | iq | Baxianyudonghanyu | Sichuan | Baxian | TNC | VI | 2 |
| 217 | iw | Zoumayanghongheyu | Sichuan | Chongqing | TNC | VI | 2 |
| 218 | ak | Laiyangyu | Shandong | Laiyang | TNC | VII | 4 |
| 219 | as | Kuanzhuangbaigengyu | Yunnan | Fumin | TNC | VII | 4 |
| 220 | dt | Wolonggangyu | Henan | Nanyang | TNC | VII | 4 |
| 221 | ed | Laiyang8520yu | Shandong | Laiyang | TNC | VII | 4 |
| 222 | el | Zaoyangyu | Hubei | Zaoyang | TNC | VII | 4 |
| 223 | hv | Wangchangcunchangyu | Hubei | Xiantao | TNC | VII | 4 |
| 224 | jo | Neijianglianhuayu | Sichuan | Neijiang | TMC | VII | 4 |
| 225 | bw | Jinshayu | Fujian | Nanping | TNC | VIII | 3 |
| 226 | by | Chiyayu | Guangdong | Guangzhou | TNC | VIII | 3 |
| 227 | ch | Changshayuhuashuiyu | Hunan | Changsha | TNC | VIII | 3 |
| 228 | co | Kunmingyu-3 | Yunnan | Kunming | TNC | VIII | 3 |
| 229 | df | Tian-21 | Hubei | Wuhan | TNC | VIII | 3 |
| 230 | dl | Haikouhongyayu | Hainan | Haikou | TNC | VIII | 3 |
| 231 | eg | Yuganyu | Jiangxi | Yugan | TNC | VIII | 3 |
| 232 | ey | Fuzhouhongyu | Fujian | Fuzhou | TNC | VIII | 3 |
| 233 | fy | Wuheyu-1 | Guangdong | Guangzhou | TNC | VIII | 3 |
| 234 | gf | Duowenhongganyu | Hainan | Duowen | TNC | VIII | 3 |

TMC: the multi-corm type, TNC: the multi-cormel type. NA：not accessible.
