# Supplementary material for: Genetic diversity and population structure of eddoe taro in China using genome-wide SNP markers
Source: PeerJ. 2020 Dec 8;8:e10485. doi: 10.7717/peerj.10485 (PMC7731653; doi:10.7717/peerj.10485)
Supplement: Supplemental Information 4 [file peerj-08-10485-s004.docx]

Table S2. **Sequencing and identified SNPs statistics of 234 taro accessions**.

| Sample code | Clean Reads | GC Percentage (%) | Q30 Percentage (%) | SLAF number | Average depth |
| --- | --- | --- | --- | --- | --- |
| aa | 5368765 | 47.06 | 93.94 | 459047 | 10.47 |
| ab | 8088955 | 45.41 | 93.51 | 438114 | 16.54 |
| ac | 5474693 | 46.64 | 93.77 | 469395 | 10.45 |
| ae | 6844739 | 46.51 | 93.21 | 412869 | 14.84 |
| af | 5720174 | 46.81 | 93.40 | 370676 | 13.78 |
| ag | 5020564 | 46.92 | 94.14 | 432410 | 10.55 |
| ah | 7122726 | 44.96 | 93.26 | 379159 | 16.87 |
| aj | 6376516 | 46.36 | 93.51 | 454893 | 12.55 |
| ak | 5705804 | 46.59 | 94.06 | 453009 | 11.34 |
| am | 7228406 | 46.02 | 92.47 | 462949 | 13.55 |
| an | 4960528 | 47.35 | 93.92 | 396907 | 11.31 |
| ao | 6088383 | 46.89 | 93.22 | 405892 | 13.37 |
| ar | 5133905 | 46.93 | 93.67 | 413443 | 11.20 |
| as | 5508080 | 47.02 | 93.76 | 479962 | 10.27 |
| au | 5784735 | 47.11 | 93.50 | 499127 | 10.33 |
| av | 4958110 | 46.84 | 92.90 | 374328 | 11.81 |
| aw | 5743005 | 46.71 | 93.31 | 452208 | 11.31 |
| ax | 5033135 | 46.92 | 93.60 | 439558 | 10.28 |
| ay | 7316141 | 46.01 | 94.06 | 385161 | 17.44 |
| az | 4875743 | 46.39 | 93.51 | 427976 | 10.20 |
| ba | 8011510 | 44.93 | 93.19 | 389499 | 18.40 |
| bb | 5560012 | 45.84 | 93.72 | 400594 | 12.56 |
| bc | 7477806 | 45.60 | 93.42 | 305545 | 22.25 |
| bd | 6184055 | 45.90 | 92.47 | 354158 | 15.36 |
| be | 6164188 | 46.12 | 93.47 | 358670 | 15.47 |
| bf | 7781293 | 46.03 | 93.82 | 384361 | 18.43 |
| bg | 8896389 | 46.12 | 94.33 | 404016 | 20.04 |
| bh | 5851764 | 45.99 | 93.32 | 366937 | 14.32 |
| bi | 9653149 | 44.16 | 92.69 | 335428 | 25.78 |
| bj | 5072702 | 46.57 | 93.76 | 358220 | 12.76 |
| bk | 4984779 | 46.37 | 93.64 | 412184 | 10.89 |
| bl | 5951555 | 46.39 | 93.14 | 349580 | 15.32 |
| bm | 4857271 | 46.26 | 93.90 | 364053 | 12.07 |
| bn | 5658067 | 45.84 | 93.41 | 316661 | 16.09 |
| bo | 5792995 | 45.62 | 93.47 | 307017 | 17.05 |
| bp | 6363538 | 45.75 | 93.67 | 419374 | 13.66 |
| bq | 7338878 | 45.88 | 93.55 | 364470 | 18.12 |
| br | 5476097 | 46.45 | 93.62 | 387783 | 12.80 |
| bs | 5753256 | 46.64 | 93.64 | 403673 | 12.93 |
| bt | 5418202 | 46.49 | 93.73 | 436942 | 11.16 |
| bu | 5410033 | 46.25 | 93.54 | 369427 | 13.24 |
| bv | 5829093 | 46.39 | 93.46 | 377374 | 14.00 |
| bw | 5120802 | 46.51 | 94.09 | 382997 | 12.11 |
| bx | 4767733 | 46.38 | 93.65 | 377618 | 11.27 |
| by | 6054482 | 45.94 | 93.75 | 374210 | 14.58 |
| bz | 4470323 | 46.40 | 93.50 | 383274 | 10.46 |
| ca | 5295523 | 46.47 | 93.86 | 441998 | 10.80 |
| cc | 5220044 | 46.69 | 93.81 | 432956 | 10.83 |
| cd | 5796652 | 46.34 | 93.65 | 394485 | 13.30 |
| ce | 5256234 | 46.58 | 93.14 | 415356 | 11.24 |
| cf | 5369075 | 46.50 | 93.70 | 442561 | 10.87 |
| cg | 7879701 | 45.86 | 93.55 | 474094 | 14.93 |
| ch | 5106664 | 46.63 | 92.83 | 335517 | 13.46 |
| ci | 5601699 | 46.24 | 93.54 | 354286 | 14.27 |
| cj | 5683027 | 46.27 | 93.65 | 384153 | 13.40 |
| ck | 5619858 | 46.19 | 94.15 | 384910 | 13.29 |
| cl | 5022781 | 45.91 | 93.54 | 423204 | 10.60 |
| cm | 4907441 | 45.91 | 93.42 | 340168 | 13.00 |
| cn | 5777347 | 46.31 | 93.55 | 460985 | 11.26 |
| co | 3975776 | 45.99 | 92.95 | 308187 | 11.40 |
| cq | 5660059 | 45.98 | 93.00 | 377297 | 13.39 |
| cr | 5130450 | 46.11 | 93.28 | 384222 | 11.87 |
| cs | 4522572 | 45.59 | 93.79 | 330846 | 12.45 |
| ct | 5704134 | 45.93 | 93.67 | 435558 | 11.77 |
| cu | 5485233 | 46.02 | 93.07 | 419764 | 11.64 |
| cv | 9631701 | 43.86 | 93.53 | 405032 | 21.28 |
| cw | 6616683 | 44.39 | 93.37 | 340898 | 17.44 |
| cx | 7725564 | 44.70 | 93.45 | 333911 | 20.82 |
| cy | 6873013 | 45.14 | 93.10 | 331910 | 18.69 |
| cz | 8140077 | 43.44 | 93.59 | 257202 | 28.84 |
| da | 9601953 | 43.07 | 93.88 | 310261 | 28.28 |
| db | 8958398 | 44.36 | 93.22 | 298280 | 27.17 |
| dc | 8528072 | 43.98 | 93.51 | 344016 | 22.45 |
| dd | 9145454 | 44.19 | 93.41 | 325486 | 25.40 |
| de | 9694558 | 43.71 | 93.27 | 333627 | 26.25 |
| df | 7447316 | 43.59 | 93.74 | 257638 | 26.26 |
| dg | 10250685 | 44.93 | 93.32 | 383111 | 24.08 |
| dh | 8553972 | 44.85 | 93.05 | 429068 | 17.74 |
| di | 8772852 | 44.64 | 93.04 | 388582 | 20.19 |
| dj | 9473466 | 44.80 | 92.97 | 334383 | 25.47 |
| dk | 9850969 | 44.70 | 93.37 | 363233 | 24.56 |
| dl | 9420055 | 44.49 | 93.17 | 334552 | 25.14 |
| dm | 9685009 | 44.13 | 93.18 | 336794 | 25.96 |
| dn | 8337618 | 44.48 | 93.40 | 318565 | 23.66 |
| do | 5516939 | 45.06 | 93.08 | 310362 | 15.93 |
| dp | 7764374 | 44.44 | 93.22 | 369520 | 18.86 |
| dq | 8661129 | 43.74 | 93.78 | 307091 | 25.79 |
| dr | 6066543 | 44.10 | 93.18 | 347472 | 15.58 |
| ds | 6362903 | 44.43 | 93.54 | 346442 | 16.63 |
| dt | 7923814 | 44.13 | 93.04 | 353495 | 20.05 |
| dv | 7944349 | 44.75 | 92.88 | 376872 | 18.76 |
| dw | 8451404 | 44.17 | 92.92 | 388278 | 19.33 |
| dx | 7031877 | 44.10 | 92.54 | 365810 | 16.91 |
| dy | 6512320 | 44.27 | 92.29 | 303692 | 18.98 |
| dz | 8333171 | 43.06 | 92.64 | 346151 | 21.28 |
| ea | 6861589 | 44.07 | 92.74 | 346357 | 17.65 |
| eb | 6987904 | 43.89 | 93.59 | 335410 | 18.74 |
| ec | 7378190 | 44.23 | 93.58 | 385457 | 17.03 |
| ed | 7038579 | 44.35 | 93.40 | 362406 | 17.44 |
| ee | 9932596 | 43.78 | 92.10 | 449479 | 18.95 |
| eg | 8323955 | 43.78 | 92.98 | 319937 | 23.33 |
| eh | 9402221 | 44.16 | 92.45 | 362714 | 23.06 |
| ei | 8017183 | 42.67 | 92.80 | 342712 | 20.83 |
| ej | 7928567 | 42.29 | 92.76 | 319357 | 22.17 |
| el | 7591983 | 42.68 | 92.96 | 339919 | 19.83 |
| em | 8867071 | 43.86 | 92.55 | 375480 | 20.90 |
| en | 7930172 | 43.56 | 92.96 | 348492 | 20.25 |
| eo | 6027310 | 43.51 | 92.59 | 320658 | 16.73 |
| ep | 6023954 | 44.77 | 92.89 | 326721 | 16.22 |
| eq | 7935019 | 44.35 | 92.66 | 423627 | 16.32 |
| er | 9281913 | 43.98 | 91.54 | 328407 | 24.91 |
| es | 7737401 | 43.76 | 92.48 | 306088 | 22.66 |
| et | 7738812 | 44.48 | 92.78 | 317885 | 21.91 |
| eu | 8229163 | 43.48 | 92.60 | 290687 | 25.49 |
| ev | 8132151 | 44.04 | 92.53 | 329840 | 21.98 |
| ew | 8057830 | 44.12 | 92.69 | 374737 | 19.09 |
| ex | 7542792 | 43.68 | 92.93 | 364657 | 18.37 |
| ey | 9069040 | 43.76 | 92.75 | 309106 | 26.16 |
| ez | 7693992 | 44.27 | 92.53 | 310475 | 22.19 |
| fa | 7214860 | 43.13 | 92.34 | 322998 | 19.56 |
| fb | 6145786 | 44.02 | 92.85 | 306244 | 17.89 |
| fc | 8640549 | 44.12 | 93.11 | 374304 | 20.67 |
| fd | 5989514 | 43.60 | 92.76 | 317636 | 16.81 |
| fe | 6459421 | 43.73 | 92.38 | 278128 | 20.75 |
| ff | 7088795 | 43.38 | 92.61 | 316775 | 19.82 |
| fg | 8250676 | 44.29 | 94.13 | 313585 | 24.21 |
| fi | 7278345 | 44.09 | 92.21 | 349494 | 18.21 |
| fj | 7648265 | 44.39 | 92.17 | 361625 | 18.58 |
| fk | 6170513 | 43.46 | 93.55 | 254715 | 21.90 |
| fl | 18152113 | 46.70 | 94.04 | 481534 | 34.55 |
| fm | 7432982 | 43.68 | 92.63 | 347319 | 19.09 |
| fn | 6452071 | 43.91 | 92.70 | 315797 | 18.06 |
| fo | 7530722 | 43.68 | 94.38 | 351798 | 19.65 |
| fp | 6312259 | 44.00 | 94.14 | 289396 | 19.96 |
| fq | 6646415 | 43.99 | 94.06 | 269801 | 22.66 |
| fr | 7104572 | 43.56 | 94.20 | 350826 | 18.47 |
| fs | 9044612 | 43.61 | 94.06 | 363594 | 22.72 |
| ft | 7039308 | 43.70 | 93.49 | 371924 | 16.98 |
| fu | 8457470 | 43.42 | 94.38 | 272404 | 28.75 |
| fv | 8721100 | 45.04 | 93.91 | 354150 | 22.43 |
| fw | 8953676 | 43.54 | 94.01 | 391631 | 20.84 |
| fx | 6511034 | 43.62 | 93.49 | 340527 | 17.18 |
| fy | 10647585 | 44.63 | 94.02 | 308853 | 31.27 |
| fz | 7257686 | 42.95 | 94.28 | 307791 | 21.60 |
| ga | 11526355 | 44.14 | 94.03 | 380630 | 27.70 |
| gb | 6617626 | 42.22 | 94.29 | 270316 | 22.51 |
| gc | 5335837 | 44.81 | 94.12 | 231112 | 21.28 |
| gd | 7280388 | 43.48 | 94.11 | 324231 | 20.53 |
| ge | 6567886 | 43.75 | 94.08 | 264835 | 22.85 |
| gf | 8173524 | 43.07 | 94.50 | 295885 | 25.28 |
| gg | 7524464 | 44.36 | 94.00 | 307259 | 22.40 |
| gh | 8395773 | 42.88 | 94.59 | 267409 | 29.05 |
| gi | 8175631 | 43.43 | 94.21 | 369498 | 20.24 |
| gj | 8742210 | 42.63 | 94.14 | 317298 | 25.27 |
| gk | 9191433 | 42.37 | 94.39 | 326380 | 25.88 |
| gl | 7642756 | 42.49 | 94.05 | 267610 | 26.21 |
| gm | 10947055 | 46.29 | 93.95 | 539469 | 18.28 |
| gn | 10721349 | 45.17 | 93.82 | 442785 | 21.95 |
| go | 11557300 | 45.53 | 93.89 | 382532 | 27.53 |
| gp | 9732641 | 44.46 | 94.09 | 385819 | 23.05 |
| gq | 8583575 | 44.45 | 94.02 | 278426 | 28.40 |
| gr | 9301225 | 44.62 | 93.94 | 374747 | 22.63 |
| gs | 8390603 | 45.50 | 94.27 | 398388 | 19.20 |
| gt | 12923128 | 44.52 | 94.02 | 351571 | 33.72 |
| gu | 10531881 | 44.76 | 93.65 | 413436 | 23.09 |
| gw | 8884608 | 44.04 | 94.16 | 306993 | 26.57 |
| gx | 8084180 | 44.16 | 94.08 | 300403 | 24.65 |
| gy | 12451335 | 44.90 | 93.42 | 377283 | 29.62 |
| gz | 8387958 | 46.26 | 93.75 | 375335 | 20.23 |
| ha | 8517921 | 45.74 | 94.00 | 481386 | 15.98 |
| hb | 9395568 | 45.64 | 93.18 | 471381 | 17.68 |
| hc | 7846594 | 45.56 | 92.08 | 426838 | 15.81 |
| hd | 4891232 | 47.53 | 94.22 | 433749 | 10.21 |
| he | 7017650 | 46.51 | 94.00 | 444585 | 14.32 |
| hg | 6567667 | 46.63 | 93.65 | 490986 | 11.93 |
| hh | 6684903 | 46.38 | 93.00 | 440879 | 13.38 |
| hi | 6477796 | 46.71 | 93.35 | 475470 | 12.08 |
| hj | 6145789 | 46.65 | 93.76 | 453766 | 12.16 |
| hk | 7304012 | 45.44 | 94.41 | 448754 | 14.86 |
| hl | 6559111 | 45.46 | 93.86 | 427620 | 13.87 |
| hm | 8679372 | 45.10 | 94.49 | 348289 | 22.83 |
| hn | 6347406 | 44.23 | 94.11 | 344824 | 16.81 |
| ho | 8025572 | 44.05 | 93.91 | 349959 | 20.90 |
| hp | 6320971 | 45.45 | 93.09 | 422320 | 13.27 |
| hq | 6780736 | 45.73 | 94.00 | 409606 | 15.10 |
| hr | 7502215 | 45.69 | 94.21 | 455377 | 15.06 |
| hs | 7022396 | 45.48 | 93.83 | 399668 | 15.96 |
| ht | 9316636 | 44.72 | 92.96 | 445215 | 18.65 |
| hu | 6964440 | 45.67 | 93.94 | 381780 | 16.56 |
| hv | 7098101 | 45.83 | 94.61 | 473623 | 13.66 |
| hw | 6837846 | 45.15 | 94.06 | 413038 | 14.99 |
| hx | 11451095 | 44.89 | 92.73 | 469097 | 21.67 |
| hy | 6695481 | 46.38 | 94.08 | 495059 | 12.23 |
| hz | 6647420 | 46.24 | 94.09 | 479849 | 12.53 |
| ia | 6372603 | 46.24 | 93.89 | 489546 | 11.71 |
| ib | 5815453 | 45.43 | 93.79 | 320716 | 16.55 |
| ic | 12137323 | 46.05 | 94.20 | 427453 | 26.12 |
| id | 11790729 | 46.27 | 93.93 | 546802 | 19.51 |
| ie | 6756836 | 46.14 | 94.03 | 423013 | 14.46 |
| if | 6453085 | 46.09 | 93.85 | 403712 | 14.47 |
| ig | 6976697 | 45.96 | 94.05 | 407241 | 15.56 |
| ih | 6522382 | 46.31 | 94.04 | 445107 | 13.26 |
| ii | 7880024 | 46.11 | 94.03 | 410700 | 17.40 |
| ij | 8125047 | 45.69 | 93.86 | 384292 | 19.15 |
| ik | 7097011 | 46.32 | 93.24 | 408767 | 15.46 |
| il | 6664346 | 46.38 | 93.91 | 420431 | 14.29 |
| im | 6668909 | 45.50 | 93.91 | 431034 | 13.99 |
| in | 6665529 | 46.46 | 92.97 | 394328 | 14.99 |
| io | 6088538 | 46.13 | 93.86 | 465997 | 11.79 |
| ip | 5614507 | 46.09 | 93.93 | 415721 | 12.09 |
| iq | 6178240 | 46.00 | 94.41 | 439981 | 12.77 |
| ir | 6506359 | 45.95 | 93.90 | 480706 | 12.18 |
| is | 6946602 | 45.98 | 93.72 | 464678 | 13.43 |
| it | 7385891 | 45.76 | 93.90 | 469928 | 14.23 |
| iu | 5472213 | 45.96 | 93.43 | 434593 | 11.20 |
| iv | 7147548 | 46.05 | 93.38 | 477748 | 13.33 |
| iw | 5509006 | 45.76 | 93.66 | 422409 | 11.75 |
| ix | 10135309 | 45.50 | 94.14 | 600960 | 15.25 |
| iy | 8792076 | 46.18 | 94.10 | 471461 | 16.89 |
| ja | 9001078 | 45.12 | 93.30 | 393214 | 20.55 |
| jb | 9765637 | 44.89 | 93.73 | 376180 | 23.49 |
| jc | 9190113 | 44.69 | 93.64 | 387998 | 21.37 |
| jd | 9864290 | 44.35 | 93.15 | 349109 | 25.36 |
| je | 8271905 | 45.24 | 93.88 | 421233 | 17.73 |
| jf | 8561119 | 44.35 | 93.65 | 384346 | 20.06 |
| jg | 8363933 | 45.76 | 93.24 | 439278 | 16.98 |
| jh | 7923885 | 45.19 | 93.14 | 394081 | 17.90 |
| ji | 7473084 | 45.17 | 93.06 | 381211 | 17.42 |
| jj | 10248652 | 45.40 | 93.57 | 453891 | 20.26 |
| jk | 8569970 | 45.13 | 93.32 | 406150 | 18.83 |
| jl | 8792961 | 44.54 | 93.20 | 381802 | 20.56 |
| jm | 9365267 | 44.77 | 93.64 | 421810 | 20.01 |
| jn | 9557849 | 44.99 | 93.28 | 385349 | 22.11 |
| jo | 10071245 | 44.65 | 93.50 | 440122 | 20.52 |
| Total | 1740915205 | 10555 | 21880 | 89182752 | 4222 |
| Average | 7439809 | 45.11 | 93.50 | 381123 | 18.04 |
| Min | 3975776 | 42.22 | 91.54 | 231112 | 10.20 |
| Max | 18152113 | 47.53 | 94.61 | 600960 | 34.55 |
